# Comparative Metagenomic and Metatranscriptomic Analysis of Hindgut Paunch Microbiota in Wood- and Dung-Feeding Higher Termites

**DOI:** 10.1371/journal.pone.0061126

**Published:** 2013-04-12

**Authors:** Shaomei He, Natalia Ivanova, Edward Kirton, Martin Allgaier, Claudia Bergin, Rudolf H. Scheffrahn, Nikos C. Kyrpides, Falk Warnecke, Susannah G. Tringe, Philip Hugenholtz

**Affiliations:** 1 Energy Biosciences Institute, University of California, Berkeley, California, United States of America; 2 US Department of Energy (DOE) Joint Genome Institute, Walnut Creek, California, United States of America; 3 Leibniz-Institute of Freshwater Ecology and Inland Fisheries, Berlin Center for Genomics in Biodiversity Research, Berlin, Germany; 4 Department of Ecology and Genetics, Uppsala University, Uppsala, Sweden; 5 Fort Lauderdale Research and Education Center, University of Florida, Davie, Florida, United States of America; 6 Australian Centre for Ecogenomics, School of Chemistry and Molecular Biosciences & Institute for Molecular Bioscience, The University of Queensland, St Lucia, Queensland, Australia; University of Osnabrueck, Germany

## Abstract

Termites effectively feed on many types of lignocellulose assisted by their gut microbial symbionts. To better understand the microbial decomposition of biomass with varied chemical profiles, it is important to determine whether termites harbor different microbial symbionts with specialized functionalities geared toward different feeding regimens. In this study, we compared the microbiota in the hindgut paunch of *Amitermes wheeleri* collected from cow dung and *Nasutitermes corniger* feeding on sound wood by 16S rRNA pyrotag, comparative metagenomic and metatranscriptomic analyses. We found that *Firmicutes* and *Spirochaetes* were the most abundant phyla in *A. wheeleri*, in contrast to *N. corniger* where *Spirochaetes* and *Fibrobacteres* dominated. Despite this community divergence, a convergence was observed for functions essential to termite biology including hydrolytic enzymes, homoacetogenesis and cell motility and chemotaxis. Overrepresented functions in *A. wheeleri* relative to *N. corniger* microbiota included hemicellulose breakdown and fixed-nitrogen utilization. By contrast, glycoside hydrolases attacking celluloses and nitrogen fixation genes were overrepresented in *N. corniger* microbiota. These observations are consistent with dietary differences in carbohydrate composition and nutrient contents, but may also reflect the phylogenetic difference between the hosts.

## Introduction

As some of the most abundant and efficient lignocellulose decomposers on the planet, termites tremendously impact lignocellulose biorecycling, and rank as one of the most important “ecosystem engineers”. Termite success is enabled by their gut microbial symbionts which participate in lignocellulose depolymerization, perform subsequent fermentation, and provide important nutrients to the hosts [1]. Unlike the phylogenetically distinct lower termites, the higher termites (family Termitidae) lack protozoan symbionts and their hindguts are highly compartmentalized with different physicochemical conditions and microbial communities to collaboratively accomplish lignocellulose degrading and fermentation functions [2], [3], [4], [5]. The third proctodeal segment, P3, also called the paunch, is the largest hindgut compartment with the highest microbial cell count and concentration of fermentation products, and therefore has been suggested to be the major microbial bioreactor in the higher termite gut [5]. Consistent with this suggestion, a metagenomic analysis of the P3 microbiota of wood-feeding *Nasutitermes* sp. workers collected in a Costa Rican rainforest revealed a rich diversity of carbohydrate active enzymes as well as genes encoding other functions important to termite biology [6], revealing the genetic potential of microbial symbionts in lignocellulose degradation.

Although termites are perceived as mainly wood consumers, particularly as pests of structural lumber in the tropical and subtropical regions, many termite species feed on other resources, such as soil, grass, and litter. Some termites, especially in the genus *Amitermes*, also opportunistically and preferentially forage on herbivore dung, such as from cattle, when it is available [7], [8]. Cow dung contains undigested fibrous plant biomass, cattle endogenous secretions and microbial debris and is thus richer in nutrients than wood. Utilization of cow dung by termites facilitates the return of nutrients contained in dung to soil, impacting ecosystem nutrient cycling and maintaining the fertility and productivity of tropical soil [9].

A largely open question is whether termites harbor different microbial symbiont populations with specialized functionalities geared towards different feeding regimens, as has been suggested for other animals [10]. Therefore the aim of this study was to determine if system-specific differences exist between hindgut paunch microbiota from higher termites with different diets. Two higher termite species from the Termitidae family, *Amitermes wheeleri* and *Nasutitermes corniger*, belonging to subfamily Termitinae and Nasutitermitinae respectively, were included in our study. *A. wheeleri* can feed on various food sources (polyphagous), including not only wood but also dead grasses, small shrubs and herbivore dung, and were shown to have a preference toward dung over mesquite wood [11]. *N. corniger* are strict wood-feeders, consuming dry, wet, or partially decayed hardwoods or softwoods [12]. *A. wheeleri* workers were collected from a solar cured cow pie within which they had hollowed out chambers. Our presumption is that these termites were foraging on cow dung at the time of sampling (namely cow dung-feeding *A. wheeleri*). The *N. corniger* sample was collected from a laboratory colony fed with dry wood (namely laboratory wood-feeding *N. corniger* to distinguish from previous Costa Rican wood-feeding *Nasutitermes* sp. [6]). As in the previous metagenomic analysis of *Nasutitermes* [6], we focused our study on the largest microbial reservoir, the P3 segment, in particular the lumen fluid fraction to avoid host nucleic acids, while acknowledging that P3 epithelium-associated microbiota and other gut segments may also be important in lignocellulose degradation and nutrient utilization. We first evaluated microbial community composition using 16S rRNA gene pyrotag analysis. We then compared community functions based on metagenomic and metatranscriptomic analyses. Our analysis on *N. corniger* corroborates and extends the findings previously obtained from Costa Rican *Nasutitermes* sp. [6], while the comparison between *A. wheeleri* and *N. corniger* highlighted a number of key differences. We discovered that the P3 lumen microbial communities are phylogenetically distinct yet perform similar core termite symbiont functions such as lignocellulose degradation and homoacetogenesis, but also have distinct functionalities reflecting dietary differences, that suggest microbial adaptations to the chemical composition of dung.

## Results and Discussion

### Microbial community composition

Pyrosequencing of PCR amplicons from the V8 region of the 16S rRNA gene (pyrotag analysis) was used for community taxonomic profiling. Archaeal sequences comprised only a tiny proportion (<0.2%) of the profiled hindgut paunch lumen communities, indicating that bacteria were the major microbial symbionts in these samples. Rarefaction curves and Shannon diversity index based on operational taxonomic units (OTUs) defined by 97% sequence identity indicated that *A. wheeleri* microbiota has about three-fold higher species richness than the laboratory *N. corniger* microbiota (**Figure S1a**). It is not clear whether this indicates an adaption of *A. wheeleri* microbial community to the higher complexity of their diet or a decrease in species diversity in *N. corniger* community caused by the laboratory rearing.

**Figure 1:**
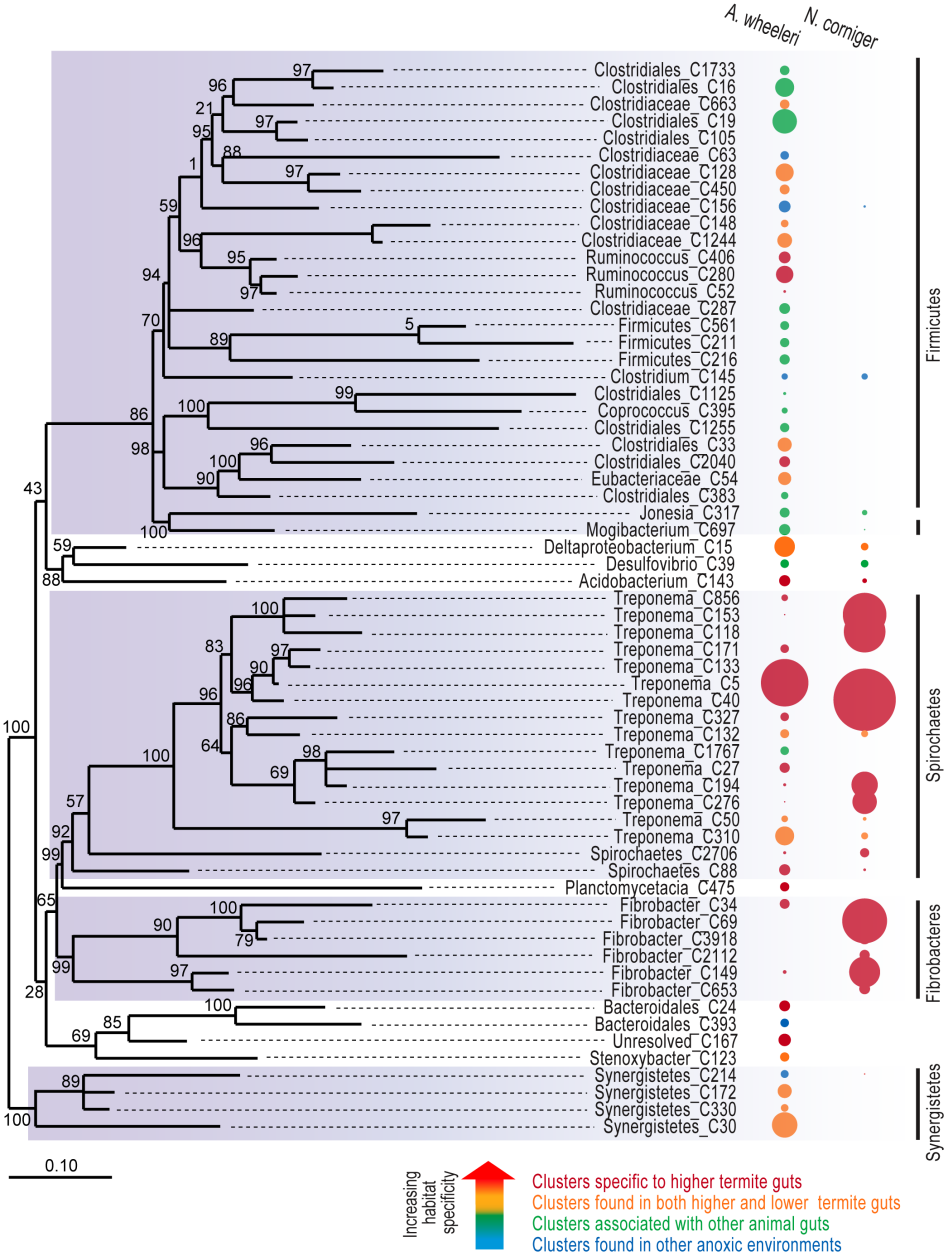
Phylogenetic affiliation, relative abundance and habitat distribution of OTUs that are >0.5% of total bacterial community. The bubble size represents the relative abundance of each OTU, and bubble color indicates the types of habitats where their closest relatives in the greengenes database were found. OTUs marked red were exclusively found in higher termites, orange OTUs were restricted to termites, including both higher and lower termites, green OTUs were also found in the guts and feces of other animals (e.g. cow, goat and elephant). Blue OTUs were found in other anoxic environments, such as anaerobic digesters.

The *N. corniger* hindgut paunch microbiota was dominated by *Spirochaetes*, particularly members of the genus *Treponema*, followed by *Fibrobacteres*, together accounting for ∼90% of the bacterial community (**Figure 1** **& Figure S1b**). Of the 16S rRNA gene pyrotag sequences classified as *Fibrobacteres*, about one third belong to *Fibrobacteres* subphylum 2 and two-thirds to Termite Group 3 (TG3); both groups having first been identified in wood-feeding higher termites (*Microcerotermes* sp. and *Nasutitermes takasagoensis*) [13], [14]. TG3 has been proposed as an independent bacterial phylum [13], however, we chose to assign this group to the *Fibrobacteres* based on its mostly consistent phylogenetic affiliation with this phylum. A genome-based analysis will be required to resolve whether TG3 is an independent phylum or a class within the *Fibrobacteres* [1]. The *Spirochetes-* and *Fibrobacteres*-dominated community profile is consistent with previous culture-independent reports of *Nasutitermes* P3 hindgut microbiota including a Costa Rican colony [6] and the same laboratory-maintained colony analyzed using a different region of the 16S rRNA gene (variable regions V3–V4) [5]. By contrast, the *A. wheeleri* hindgut largely comprised members of the *Firmicutes* phylum (47%), especially *Clostridia*, followed by *Spirochaetes* (25%). *Synergistetes* and *Deltaproteobacteria* were also found at moderate abundance (**Figure 1** **& Figure S1b**). *Fibrobacteres* (including Subphylum 2 and TG3) were only detected at a very low abundance (∼1%) in *A. wheeleri*. This is consistent with a previous suggestion that these lineages are only abundant in wood-feeding higher termites, as they are largely absent in other feeding guilds of higher termites and in lower termites [14], suggesting that both host phylogeny and diet play a role in determining *Fibrobacteres*/TG3 distribution in termite hindguts.

A potential diet-associated difference in bacterial community composition has also previously been noted between two termite species [15]: a wood-feeding lower termite, *Reticulitermes speratus*, dominated by *Spirochaetes*, mostly belonging to the genus *Treponema* [16], and a soil-feeding higher termite, *Cubitermes*, dominated by *Firmicutes*, mostly belonging to the class *Clostridia* [17]. Like cow dung, the litter-derived lignocellulose-containing soil is rich in nitrogenous compounds of microbial origin. The enrichment of *Clostridia* in two higher termite genera on relatively nitrogen-rich diets and enrichment of treponemes in two distantly related termite genera on nitrogen-poor diets supports the hypothesis that the observed community dissimilarity in our study can be partly attributed to the difference in dietary nutrients. However, an influence of host phylogeny on hindgut community structure should not be ruled out. A number of previous studies have revealed correlations between termite gut microbiota and their hosts, indicating an important role for co-evolution in the make-up of symbiont microbial communities [13], [14], [18]. It is very likely that both host phylogeny and diet influence termite symbionts, however their individual contributions cannot be explicitly resolved due to the limited number of termite species and host-diet combinations investigated in our study.

Habitat distribution of the termite gut OTUs can provide clues about the evolutionary acquisition of different microbial species. Habitat distribution was estimated for each OTU based on publicly available 16S rRNA gene sequences belonging to that OTU. Most *Treponema* and *Fibrobacter-*like OTUs characteristic of wood-feeding species have only been identified in termite guts, and are phylogenetically distinct from other members of the *Spirochaetes* and *Fibrobacteres* phyla found in different environments. This supports a previously suggested long-term co-evolution of these species with their insect hosts with increasing specialization towards lignocellulose degradation [19], [20]. By contrast, *Firmicutes* OTUs enriched in polyphagous *A. wheeleri* were also represented in other anoxic environments, many of which are associated with animal guts and feces, suggesting that some of these microbial populations may have been more recently acquired by termites in response to changes in host diet (see below).

### Gene-centric analysis of community metabolism

We performed metagenomic analyses in order to discover functional differences between the *A. wheeleri* and *N. corniger* P3 lumen microbiota. Metagenome data were generated with a combination of Sanger and 454 pyrosequencing; **Table S1** lists the summary results of metagenome sequencing, assembly and annotation. Phylogenetic composition of metagenomes was evaluated by sequence homology-based taxonomic assignment of genes belonging to a total of 38 phylogenetic marker gene families (COGs, listed in **Table S2**) (**Figure S1c**) and by all metagenome genes with function prediction (**Figure S1d**) respectively. Archaeal sequences accounted for less than 0.1% of these genes in agreement with the pyrotag data. At the phylum level, bacterial community profiles estimated using marker COGs were largely consistent with the pyrotag profiles (**Figure S1b**).

We conducted gene-centric analysis [21] to compare the metagenomes based on functional units of pfam, COG, or COG functional categories. We included the Costa Rican wood-feeding *Nasutitermes* metagenome [6] as a reference dataset in the analysis with the expectation that it would functionally resemble the laboratory-maintained *N. corniger* and help to highlight significant metabolic differences with the dung-feeding *A. wheeleri* metagenome. On a global scale, the three termite P3 metagenomes shared higher similarities in functional profiles to each other than to other animal guts or lignocellulose-degrading ecosystems, with the two wood-feeding *Nasutitermes* metagenomes exhibiting the highest similarity (**Figure S2**) despite the different sequencing depths and technologies used in these studies. Many consistencies among the three termite P3 metagenomes were also observed as compared to other metagenomes and genomes in IMG/M (**Figure S3a, b**), indicating functional commonalities in the termite hindgut paunch microbiota. In particular, cell motility and associated chemotaxis are overrepresented which are required by microorganisms to respond to steep physicochemical gradients found in the termite hindgut [2], [3], [4], [5], [22].

We next analyzed the similarities and differences in expression profiles of the *A. wheeleri* and laboratory-maintained *N. corniger* termite P3 microbiomes using metatranscriptomics. To recover expressed genes that may not have been detected in the metagenomes, we performed a *de novo* assembly of metatranscriptome reads, which was then co-assembled with the metagenome to generate a more complete reference to map cDNA reads for gene expression (**Figure S4**). A summary of metatranscriptome sequencing, *de novo* assembly, annotation and read mapping results is presented in **Table S3**. Sequence homology-based taxonomic assignment of functional genes from metatranscriptome assemblies (**Figure S1e**) indicates that the most abundant transcripts were from *Firmicutes* and *Spirochaetes* for *A. wheeleri* and *N. corniger* respectively consistent with their pyrotag and metagenome profiles (**Figure S1b,c,d**). The transcript distribution among COG categories supports the importance of cell motility and carbohydrate transport and metabolism in the *A. wheeleri* and *N. corniger* P3 microbiota as they were the most highly expressed functional categories (**Figure S5**).

**Figure 2:**
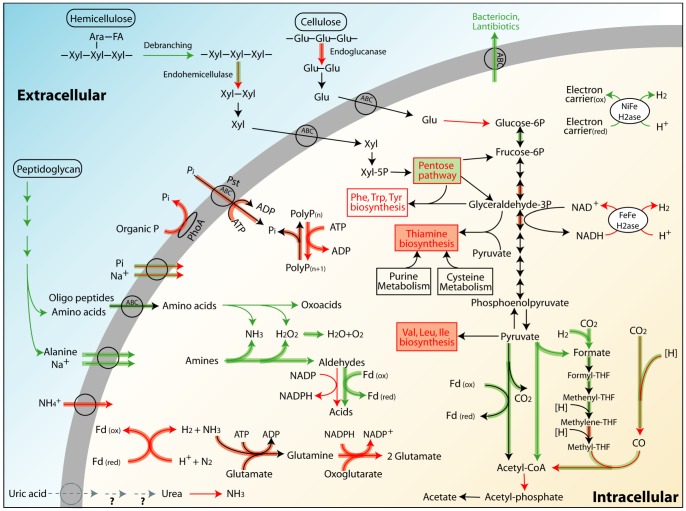
Key metabolic differences between cow dung- and wood-feeding termites based on gene and transcript abundance profiles. In this schematic summary, intracellular and extracellular reactions are separated by a cell membrane, but these reactions do not necessarily all occur in one cell. Green or red thin lines (including rectangle outlines) indicate genes more abundant in the *A. wheeleri* or *N. corniger* metagenome, respectively. Green or red thick lines (including rectangle highlights) indicate transcripts more abundant in the *A. wheeleri* or *N. corniger* metatranscriptome, respectively. Black lines indicate equal representation in both termite genera.

More specific individual functions and pathways that are differentially represented between the *A. wheeleri* and *N. corniger* metagenomes (**Table S4**) and between their metatranscriptomes (**Table S5**) were identified. The key metabolic differences are schematically presented in **Figure 2** and discussed hereafter.

### Lignocellulose degradation

Cellulose, hemicellulose and lignin are the three major components of plant cell walls. In higher termites, both hosts and their gut symbionts participate in lignocellulose degradation [1]. However in our study we only focused on the contribution of symbiont microbial communities. The previous metagenomic study of the P3 lumen fluid of *Nasutitermes* did not identify lignin degradation genes [6]. Similarly, our current analyses did not find genes from known lignin degradation pathways in the P3 microbiota of either termite species. This may be partly due to the oxygen status in P3 (microoxic periphery around an anoxic center [2], [5], [22]), which may not support the known aerobic lignin degradation pathways. Early reports of enzymatic lignin degradation during the passage through the termite gut were somewhat ambiguous (see reviews [1], [23]). Since celluloses and hemicelluloses need to be disassociated from lignin to increase accessibility for efficient enzymatic degradation, lignin modification or partial degradation (if not complete mineralization) is expected, and this functionality may be provided by gut segments not investigated in our study or by the termite host. For example, the P1 segment has a high alkalinity in several higher termite species [2], [3], [5], and alkaline pretreatment disrupts lignin structure in industrial lignocellulose degradation [24]. Further, microbial community composition varies among different compartments of higher termites [17], [25], [26], and specifically for *N. corniger*, Köhler et al. [5] have demonstrated notable differences in microbial communities among different gut segments. Therefore, other gut segments, particularly the more aerobic regions, may harbor populations capable of lignin degradation. In addition, the contribution of higher termite host to lignin degradation, although still remain unclear, cannot be ruled out.

In contrast to the absence of identifiable lignin degradation genes, glycoside hydrolases (GHs), necessary for cellulose and hemicellulose degradation, were highly represented and expressed in the termite hindguts (**Table S6**). The relative abundance of GH families in the P3 metagenomes is listed in **Table 1** according to their broad functional categories. The screening of a metagenomic fosmid library for lignocellulolytic activities identified GH5, 8, 10 and 11 gene families from a wood-feeding higher termite (*Microcerotermes* sp.) hindgut microbial community [27]. Our wood-feeding *N. corniger* metagenome also had abundant genes in these GH families, and compared to *A. wheeleri*, *N. corniger* had higher relative abundances of cellulases (GH5, 9 and 45) and endohemicellulases (GH 8, 10, 11, 26 and 53) which attack celluloses and main chains of hemicelluloses (**Table S4**). A cluster heatmap based on the abundance of individual GHs relative to total GHs showed that the two *Nasutitermes* spp. shared the highest similarity in GH profile; and the three termite P3 metagenomes exhibited higher similarities to each other than to other lignocellulose-degrading systems, including a compost adapted to switchgrass [28], tammar wallaby foregut [29], wild panda gut [30] and cow rumen [31], [32] (**Figure S6**). Interestingly, microbiota associated with switchgrass incubated in a cow rumen [32] had a high similarity in GH profile to *A. wheeleri*, while showing a lower similarity to cow rumen fed with forage grass and legume [31]. In addition to the differences in sequencing platform and efforts, and in substrates (switchgrass versus forage grass and legume), the discrepancy might be partly due to the long incubation time (72 hrs) of switchgrass, which may allow the establishment of microbes capable of attacking more recalcitrant backbones in the diet, compared to the initial colonization of fiber that preferentially used more easily accessible sugars in the side chains [31].

**Table 1:** Inventory of putative glycoside hydrolases (GHs) in plant cell wall degradation in termite hindguts and other environments^a^.

CAZy family	pfam domain	Known activities	*A. wheeleri* (Cow dung)	*N. corniger* (Lab colony)	*Nasuti-termes* sp. (Costa Rica)^b^	Compost^c^	Tammar wallaby foregut^d^	Cow rumen^e^
**Cellulases**								
GH5^*^ g	PF00150	cellulase	10.4	17.2	13.9	3.2	3.7	1.0
GH6	PF01341	endoglucanase	0	0	0	2.1	0	0
GH7	PF00840	endoglucanase	0	0	0	0.1	0	0
GH9^*^	PF00759	endoglucanase	4.2	7.0	4.3	4.3	0	0.9
GH44	BLAST search^f^	endoglucanase	0.3	0	0.8	0.4	0	0
GH45	PF02015	endoglucanase	0.8	3.6	0.6	0	0	0
GH48	PF02011	endo-processive cellulase	0	0	0	0.5	0	0
**Subtotal (%)**			**15.7**	**27.8**	**19.6**	**10.6**	**3.7**	**1.9**
**Endohemicellulase**								
GH8^*^	PF01270	endo-xylanase	1.5	4.0	2.7	0.5	0.4	0.5
GH10	PF00331	endo-1,4-β-xylanase	9.0	10.5	9.9	8.9	4.1	1.0
GH11	PF0457	xylanase	4.4	4.7	1.9	1.4	0	0.1
GH12	PF01670	endoglucanase & xyloglucan hydrolysis	0	0	0	0.6	0	0
GH26^*^	PF02156	β-mannanase & xylanase	1.4	2.9	2.0	1.5	1.9	0.8
GH28	PF00295	galacturonases	1.1	0.2	1.4	0.9	0.7	0.6
GH53^*^	PF07745	endo-1,4-β-galactanase	0.7	3.2	2.2	0.2	3.3	2.7
**Subtotal (%)**			**18.1**	**25.5**	**20.1**	**14.0**	**10.4**	**5.7**
**Cell wall elongation**								
GH16	PF00722	xyloglucanases & xyloglycosyltransferases	0.7	0.5	0.6	2.0	1.5	0.1
GH17	PF00332	1,3-β-glucosidases	0	0	0	0.1	0	0
GH74	BLAST search	endoglucanases & xyloglucanases	0.8	1.4	0.7	1.6	0.4	0
GH81	PF03639	1,3-β-glucanase	0	0	0	0.3	0	0
**Subtotal (%)**			**1.5**	**1.9**	**1.3**	**4.0**	**1.9**	**0.1**
**Debranching enzymes**								
GH51^**^	BLAST search	α-L-arabinofuranosidase	4.1	0.8	2.0	7.8	4.5	9.9
GH54	PF09206	α-L-arabinofuranosidase	0	0	0	0	0	0.2
GH62	PF03664	α-L-arabinofuranosidase	0	0	0	1.7	0	0
GH67	PF07477, 07488, 03648	α-glucuronidase	2.5	2.0	3.3	3.6	1.9	0
GH78^**^	PF05592	α-L-rhamnosidase	1.9	0.4	0.8	8.1	9.3	5.1
**Subtotal (%)**			**8.5**	**3.2**	**6.1**	**21.2**	**15.6**	**15.2**
**Oligosaccharide-degrading enzymes**								
GH1	PF00232	β-glucosidase & other β-linked dimers	2.8	3.1	2.5	9.2	22.7	1.8
GH2^**^	PF00703, 02836, 02837	β-galactosidases & other β-linked dimers	15.7	7.1	13.6	8.6	8.9	28.5
GH3^**^	PF00933, 01915	mainly β-glucosidases	17.4	15.1	15.5	12.2	26.8	26.6
GH29^**^	PF01120	α-L-fucosidase	3.3	1.8	1.2	2.1	0.7	4.2
GH35	PF01301	β-galactosidases & other β-linked dimers	0.1	0.1	0.6	0.6	1.1	1.9
GH38	PF01074, 07748	α-mannosidase	2.1	1.7	4.2	2.6	1.1	2.6
GH39	PF01229	β-xylosidase	1.6	1.4	1.5	1.0	0.4	0.3
GH42^*^	PF02449, 08533, 08532	β-galactosidase	2.8	5.0	6.9	2.5	3.0	1.9
GH43^**^	PF04616	arabinases & xylosidases	10.1	5.2	6.6	11.3	3.7	9.3
GH52	PF03512	β-xylosidase	0	1.1	0.3	0	0	0
**Subtotal (%)**			**55.9**	**41.6**	**52.9**	**50.1**	**68.4**	**77.1**

a The listed value is the population abundance weighted relative abundance (%) of GH families among the total GHs included in the table used in Allgaier *et al* [28].

b An improved version of assembly was used, leading to subtle differences in numbers from originally reported by Warnecke *et al* [6].

c Percentages of GHs were directly from Allgaier *et al* [28].

d GH gene counts were from Pope *et al* [29]and the percentages were renormalized by total number of GHs included in the table used in Allgaier *et al* [28], for comparison among these studies.

e Percentages were calculated based on GH gene counts reported in Brulc *et al* [31].

f No pfam domain is available and the identification is performed by BLAST search.

g GHs over- (^**^) or under-represented (^*^) in *A. wheeleri* compared to the laboratory *N. corniger* (after adjusting for multiple hypothesis test, with a false discovery rate P-value cutoff of 0.05), and the difference was supported by the comparison between *A. wheeleri* and Costa Rican *Nasutitermes* sp.. The comparison was based on GH abundances normalized by the total abundance of GHs listed in this table.

Debranching enzymes that cleave side chains of hemicelluloses were overrepresented in *A. wheeleri* as compared to *N. corniger* metagenomes. The most abundant debranching enzymes in *A. wheeleri* were arabinofuranosidases of GH family 51, which catalyze the hydrolysis of α-1,2 and α-1,3-arabinofuranosidic bonds in arabinose-containing side chains of hemicelluloses, such as (glucurono)arabinoxylans, the major form of hemicellulose in grasses but not wood [33]. Arabinofuranosidases and rhamnosidases (GH78) were also abundant in grass-decomposing systems: switchgrass-adapted compost [28], cow rumen [31], [32] and tammar wallaby foregut [29], suggesting that these enzymes are associated with grass hemicelluloses. As the cattle at the sampling site primarily consumed range grasses, the diet of *A. wheeleri* is ultimately derived from grass. In general, grass has a higher hemicellulose content than wood, and the major form is (glucurono)arabinoxylans, compared to xyloglucan and glucuronoxylan that dominate in wood [33], [34]. Therefore, the higher abundances of these two GHs (51 and 78) in *A. wheeleri* than in the two *Nasutitermes* species is consistent with the grass-origin of cow dung and/or the polyphagous nature of *A. wheeleri* that also feed on grass. Overall, these observations support the inference that diet, specifically carbohydrate composition, drives the GH profile in the lignocellulose-degrading gut environments [31].

In the metatranscriptomes, carbohydrate transport and metabolism was the second most highly expressed COG function category for both termites (**Figure S5**). Although a wide array of GHs was identified in metagenomes, not all of these GHs were detected in the metatranscriptomes (**Figure S7**), in part due to the low recovery of functional genes in metatranscriptomes as compared to rRNA (**Table S3**), but also likely linked to lower expression of these genes, highlighting the limitation of solely relying on metagenomes to infer community functions. In particular, the expression of debranching enzymes and oligosaccharide degrading enzymes was largely undetected, compared to enzymes targeting celluloses and main chains of hemicelluloses. GH5 (cellulases) and GH11 (xylanases) had the highest expression levels among all GHs in *N. corniger* and *A. wheeleri* metatranscriptomes respectively. Compared to wood, grass in general has a higher content of xylan [34], consistent with the high expression of xylanase genes in *A. wheeleri*. Notably, GH11 was not the most abundant GH in either metagenome, and the top ten highly expressed GH11 genes were identified solely from the *de novo* assembled metatranscriptome. By homology, seven of those ten can be assigned to *Firmicutes*. Without including the *de novo* assembled metatranscriptome in the reference for mapping the metatranscriptome reads, we would have overlooked the expression of these genes. This highlights the value of *de novo* metatranscriptome assembly in retrieving highly expressed genes that are present at low relative abundance in metagenomes.

Cohesins and dockerins, key components of cellulosomes, were detected in *A. wheeleri* while being largely undetected in both *Nasutitermes* species. These cohesin and dockerin genes were most similar to those from *Clostridia*, many species of which are known cellulosome producers, and this is consistent with the dominance of *Clostridia* in *A. wheeleri*. By contrast, a protein family TIGR02145, markedly abundant in Costa Rican *Nasutitermes* sp. [6], was also abundant in the laboratory *N. corniger* but much less so in *A. wheeleri*. This family primarily consists of *Fibrobacter*-specific extracytoplasmic proteins, and is hypothesized to be an analog of cohesins [6]. The different abundances of these protein families primarily reflect the difference in microbial community composition, and suggest that a similar function (substrate binding in this case) was provided by different microbial populations.

### Nitrogen metabolism and dietary nutrient availability

Previously a rich array of nitrogen fixation genes was discovered in Costa Rican *Nasutitermes* symbionts [6], and we also observed large numbers of genes encoding nitrogenase components in the laboratory *N. corniger*. However, some of these genes were strikingly underrepresented in the *A. wheeleri* hindgut metagenome, although still over-represented compared to other ecosystems, such as switchgrass-adapted compost [28] and farm soil [21]. Furthermore, higher gene expression of nitrogen fixation and ammonia assimilation pathways was also observed in *N. corniger* as compared to *A. wheeleri*. This suggests that in addition to fixing nitrogen, the *A. wheeleri* hindgut community utilizes fixed nitrogen from the diet. In cow dung, total nitrogen is usually 1–2% by dry weight [35], [36] and mainly present in organic forms (e.g. crude protein content of 6–10% [37]), in contrast to wood, which is extremely poor in nitrogen (e.g. total nitrogen as low as 0.05% [38]). Apart from the undigested lignocellulose, cow dung contains endogenous secretions (such as mucus) and microbial cells and debris (mainly bacterial cell walls) from the cattle gastrointestinal tract [39], or from microbial decomposers of dung after its release, all of which can be potential sources of combined nitrogen. Consistent with usage of such compounds, we observed a higher abundance of genes involved in the degradation and utilization of peptidoglycan, a major component of microbial cell walls, in *A. wheeleri* relative to *N. corniger* (**Figure 2**, **Table S4**). Similarly, the soil-feeding termite *Cubitermes orthognathus* has been shown to metabolize peptidoglycan and other microbial cell components at a higher rate than cellulose [40]. Soil, like dung, has higher levels of fixed nitrogen than typical lignocellulose termite diets [41]. In addition, a number of gene families involved in amino acid and oligopeptide transport had higher relative expression in *A. wheeleri*, as did genes associated with amino acid and amine oxidation (**Figure 2**). Correspondingly, biosynthesis pathways for a number of amino acids were underrepresented in *A. wheeleri* as compared to *N. corniger*, including valine, leucine, isoleucine, phenylalanine, tryptophan and tyrosine, several of which are abundant amino acids in cow dung [42].

Notably, one of the enriched phyla in *A. wheeleri* is *Synergistetes*, accounting for ∼8% of total bacterial 16S rRNA gene pyrotags, compared to 0.1% in *N. corniger*; its augmentation in *A. wheeleri* is also supported by metagenome and metatranscriptome data (**Figure S1**). The cultivated representatives of the *Synergistetes* phylum generate energy primarily through peptide/amino acid fermentation, and the majority of them cannot grow on carbohydrates [43]. The available *Synergistetes* genomes are particularly rich in genes for COG F, amino acid transport and metabolism (an average of ∼11% of genes belonging to COG F among all COG categories) compared to other bacterial phyla, especially *Spirochaetes* (5% for COG F) [44]. In addition, most *Synergistetes* genomes also lack nitrogenases for fixing nitrogen. Therefore the augmentation of *Synergistetes* in dung-feeding *A. wheeleri* is also consistent with the higher protein content in cow dung.

Further evidence reflecting the higher nitrogen content in the diet of *A. wheeleri* is a paucity of genes involved in the uric acid cycle. When dietary nitrogen becomes limiting, termite bacterial symbionts can recycle uric acid, a host metabolic waste, to generate ammonia for assimilation [45]. In our study, although neither uricase, nor other enzymes participating in the aerobic degradation of uric acid to urea was identified in any of the three metagenomes, ureases were found in the two *Nasutitermes* species and not in *A. wheeleri* (**Figure 2**). In addition, we observed higher abundance and expression of ammonium transporters in the laboratory *N. corniger* than in *A. wheeleri* (**Figure 2**). In model bacteria, ammonia transporters were expressed in response to nitrogen limitation, and the transportation was turned off when nitrogen is sufficient and cellular glutamine concentration is high [46], [47]. Therefore, the overrepresentation and higher expression of ammonia transporters in *N. corniger* also likely indicated dietary nitrogen limitation. Thus, hindgut microbiota may play a larger role in nitrogen metabolism in wood-feeding *N. corniger* than in dung-feeding *A. wheeleri* as suggested by the overrepresentation of diverse functions related to nitrogen metabolism in *N. corniger*.

The lower nutrient content of wood relative to cow dung was also reflected by the overrepresentation of vitamin biosynthesis pathways (particularly thiamine) in the *N. corniger* metagenome and metatranscriptome (**Figure 2**). Wood has a lower phosphorus content (∼0.01% [48]) than cow dung (e.g. 0.55% by dry weight [49]). The *N. corniger* hindgut had a higher abundance of alkaline phosphatase and polyphosphate kinase genes, and higher expression levels of these genes and high affinity phosphate transporters than *A. wheeleri*, consistent with phosphorus limitation in the wood-feeding gut environment. Taken together, the community functional differences associated with higher availabilities of nutrients may explain why some termite genera preferentially feed on cow dung despite the enrichment for more recalcitrant lignocellulosic components after passage through the cattle gastrointestinal tract. This feeding behavior plays an important role in facilitating nutrient recycling and fertilizing of top soil in (sub)tropical regions [9].

### Hydrogenases

**Figure 3:**
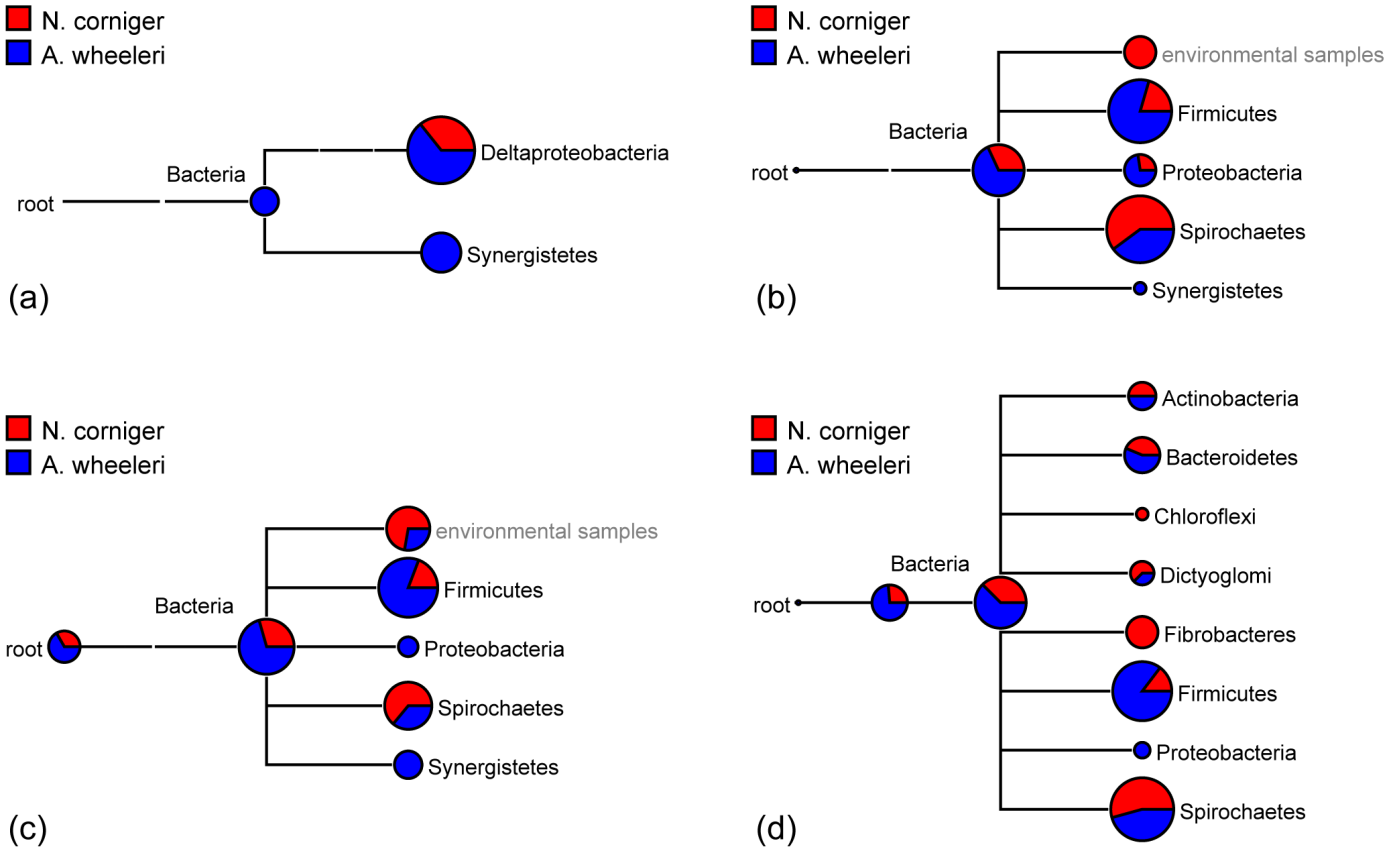
Taxonomic assignment of nickel-iron (NiFe) hydrogenases (a), the large subunit of iron-only (FeFe) hydrogenases (b), formyl tetrahydrofolate synthase (FTHFS) (c), and glycoside hydrolases family 3 (GH3) (d) by MEGAN using Blastp results against the NR database.

Hydrogen is a key fermentation product and intermediate in the termite hindgut microbial food chain [50], [51], and can be generated and consumed through nickel-iron (NiFe) hydrogenase or iron-only (FeFe) hydrogenase activities. NiFe hydrogenase sequences were largely absent from both the *N. corniger* metagenome and metatranscriptome, and were found at low abundance in the *A. wheeleri* metagenome and metatranscriptome. Based on sequence similarity, NiFe hydrogenases were identified as belonging to members of the *Synergistetes* and *Deltaproteobacteria* (**Figure 3a**), both of which were minor constituents in *N. corniger* and were augmented in *A. wheeleri*. The FeFe hydrogenase genes were abundant in all three termite metagenomes, although most abundant in *N. corniger*. Their transcripts were found in much higher levels relative to NiFe hydrogenase transcripts, but were not statistically different between the two termite metatranscriptomes. The protein sequence homology-based phylogenetic classification of the FeFe hydrogenase large subunits showed that these genes were predominantly contributed by *Spirochaetes* in *N. corniger*, in contrast to *A. wheeleri*, where the largest fraction of these genes was present in *Firmicutes* (**Figure 3b**).

### Homoacetogenesis

Homoacetogenesis, CO_2_ reduction to form acetate, is the major H_2_ sink in wood-feeding termites compared to methanogenesis in some soil-feeders [52], [53]. Homoacetogenesis is also likely the major H_2_ sink for the dung-feeding *A. wheeleri*, as evidenced by the abundance of homoacetogenesis genes and paucity of Archaea and methanogenesis genes. It was previously observed that NADPH-dependent formate dehydrogenases, which catalyze the first step in synthesizing the methyl group of acetate, were rare in Costa Rican *Nasutitermes* sp. [6] conflicting with the high abundances of other genes in the homoacetogenesis pathway. One previously hypothesized explanation for this anomaly was that an alternative route was used to generate formate, such as through the reaction of pyruvate formate lyases [6]. This protein family was present in both *A. wheeleri* and *N. corniger*, at a higher abundance and expression level in the former (**Figure 2**). Alternatively, cysteine and selenocysteine variants of hydrogenase-linked formate dehydrogenases (FDH(H)) that use H_2_ instead of NADPH were identified in the genome of *Treponema primitia*, a termite gut isolate, and were suggested to be an adaptation to the H_2_-rich termite gut [54]. The FDH(H) genes were also detected in a number of wood-feeding termite and cockroach guts, suggesting the importance of FDH(H) in CO_2_-reductive acetogenesis in these gut environments [55]. Homologs of these formate dehydrogenases were present in both *A. wheeleri* and *N. corniger* metagenomes, and were more abundant in the *A. wheeleri* metagenome. In addition, H_2_-formate dehydrogenases and several other genes associated with this pathway were expressed at higher levels in *A. wheeleri* relative to *N. corniger* (**Figure 2**).

Formyl tetrahydrofolate synthase (FTHFS), a key enzyme in the homoacetogenic pathway, has been used as a functional and phylogenetic marker for homoacetogenic bacteria [1]. Protein sequence similarity based taxonomic assignment indicated the dominant FTHFS shifted from *Spirochaetes* in *N. corniger* to *Firmicutes* in *A. wheeleri* (**Figure S3c**). Similar shifts were also observed for hydrogenases as discussed earlier, and glycoside hydrolase family 3 (**Figure 3d**), an abundant GH family in termite metagenomes for oligosaccharide degradation. These data indicate that key functional niches in the termite hindgut (e.g. oligosaccharide degradation, hydrogen formation and homoacetogenesis) are maintained through the activities of phylogenetically distinct populations in *A. wheeleri* and *N. corniger* as has been previously noted for hydrogenases [56] and FTHFS [57] in a range of termite genera. Interestingly, it was previously suggested that termite symbiotic *Spirochaetes* may have acquired their CO_2_ reductive acetogenesis capability through lateral gene transfer from *Firmicutes*, although whether the gene transfer event occurred before or after *Spirochaetes* had initially become termite symbionts has not been determined [58]. In the study by Ottesen and Leadbetter [57], treponeme-like sequences dominated the FTHFS sequences recovered from wood-feeding cockroaches, lower and higher termites; contrasting to the abundance of *Firmicutes* FTHFS sequences from omnivorous cockroaches and higher termites with other feeding regimes, including litter and grass/soil. This also supports our hypothesis that host diet plays an important role in determining the population occupying a niche.

### Cell motility and chemotaxis

Termite guts exhibit steep physicochemical gradients [2], [3], [4], [5], [22]. Motility enables termite microbial symbionts actively accessing their substrates and locating themselves at a physiochemical gradient thermodynamically favorable for their reactions. In our study, motility-related genes are overrepresented in both termite P3 metagenomes compared to averaged data from a range of other microbiomes (**Figure S3**) and were also highly expressed by both termite communities (**Figure S5**). Among the termites, we observed higher abundances of the cell motility and chemotaxis genes in *N. corniger* (**Table S4**). Particularly, a methyl-accepting chemotaxis protein (MCP) family was notably more abundant in *N. corniger*. As all major microbial phyla in both termites are motile, we hypothesize that the difference in cell motility and chemotaxis gene abundance primarily reflects community compositional differences, in particular, the relative abundance of *Spirochaetes*. Often known as spiral-shaped bacteria, *Spirochaetes* are excellent swimmers with a unique form of motility in highly viscous liquids, such as termite lumen fluid. Their sheathed flagella reside within the periplasmic space, and are triggered by methylation or demethylation of MCPs to rotate [59]. Their motility accelerates as media viscosity increases, while most bacteria with external flagella are slowed down or stopped [60]. This gives *Spirochaetes* an advantage in the highly viscous termite hindgut environment by allowing them to penetrate substrates and locate themselves at physicochemical gradients favorable for their biochemical reactions. A redundancy of chemotaxis genes [61] and numerous MCP genes [62] have been found in some *Spirochaetes* isolate genomes. Consistent with this observation, the majority of MCP genes identified in the present study were classified as belonging to *Spirochaetes*, even in *A. wheeleri* where *Spirochaetes* are not the most abundant phylum (**Figure S8**). This likely reflects a higher number of MCP genes per genome in the termite *Spirochaetes* populations relative to other motile bacterial populations and also accounts for the higher abundance of MCP genes in *N. corniger* metagenome (**Table S4**).

### Adaptation to dung

Despite being a rich source of nutrients, cow dung may impose a competitive pressure on termite gut microbiota due to the presence of microbial cells and their products in ingested dung. Consistent with this idea is a higher abundance of defense genes in *A. wheeleri* than in *N. corniger*. Specifically, we observed a higher abundance and expression of genes in *A. wheeleri* associated with export of antimicrobial peptides (**Figure 2** **, Tables S4 & S5**), such as bacteriocin, generally found in Gram-positive bacteria for use against closely related bacteria. *A. wheeleri* P3 microbiota also exhibited higher abundances of genes involved in resistance to antimicrobial compounds (**Table S4**), probably indicating higher levels of these compounds in cow dung relative to wood, as antimicrobial compounds can be naturally produced by rumen symbionts (and obtained through cattle food additives to enhance beef production) [63].

However, on an evolutionary time scale, the microbial competitors present in dung may have also contributed to the hindgut paunch microbiota, either via lateral gene transfer, or whole organism transfer. Close relatives of some *Firmicutes* OTUs in *A. wheeleri* have been found in other anoxic environments, e.g. animal guts or dung (**Figure 1**). *Firmicutes* is the most abundant phylum in the cow rumen [31] and many *Firmicutes* species are able to form endospores. In line with this, we saw higher abundances of genes related to sporulation and germination in *A. wheeleri* (**Table S4**), and this capability may have allowed rumen-derived *Firmicutes* to survive passage through the termite gut facilitating colonization of the hindgut paunch and/or lateral gene transfer.

In summary, comparison of P3 lumen microbiota from two different genera of higher termites with different diets revealed distinct community and functional profiles strongly reflecting dietary differences, but also likely influenced by termite host phylogeny. Despite these differences, functions essential to termite biology were conserved in both P3 microbiomes. The relative contributions of host phylogeny and diet will need to be addressed by molecular analyses of additional termite taxa, including congeneric species with different feeding habits. The current study provides a useful baseline for such future work as the genera *Nasutitermes* and *Amitermes* both comprise numerous species with varied diets [9], [64].

## Materials and Methods

### Termite sources and DNA extraction

The *Amitermes wheeleri* sample was collected from a cow pie in the Arizona desert (31.533, −110.012, 4607 ft), primarily vegetated with creosote bushes and grasses. *Nasutitermes corniger* was obtained from a field-collected colony in Dania Beach, Florida, and was maintained in the laboratory with *Schinus terebinthifolius* (Brazilian pepper) wood and damp cellulose sponges. The field studies did not involve endangered or protected species, and no specific permits were required for the described field studies. The hindgut P3 segment of worker termites was incised, and disrupted with 23¼ gauge syringe needles. Approximately 10 µl of phosphate buffered saline (PBS, pH 7.2) was added to the disrupted P3 and mixed by repetitive pipetting. The PBS mixed luminal contents were aspirated into a pipette and pooled into a microcentrifuge tube. The luminal contents were pooled from ∼80 worker termites from *A. wheeleri* or *N. corniger* and DNA was extracted using Cetyltrimethyl Ammonium Bromide (CTAB)-containing lysis buffer, with bead beating in the presence of phenol/chloroform/isoamylalcohol, followed by phase separation and purification with polyethylene glycol (PEG), with details described elsewhere [28].

### Pyrotag analysis of microbial community composition

Pyrotag sequencing of 16S rRNA amplicons was used for microbial community composition analysis. The experimental procedures were previously described [65]. The primer set 926F (5′-AAACTYAAAKGAATTGACGG-3′) and 1392R (5′-ACGGGCGGTGTGTRC-3′) which broadly targets Bacteria and Archaea was used to amplify the V8 regions of community 16S rRNA genes. The primers incorporated 454 adapters and barcodes so that they could be pooled and submitted for Roche 454 GS FLX sequencing. The sequences were analyzed using Pyrotagger (http://pyrotagger.jgi-psf.org) and were quality trimmed to 200 bp, clustered into operational taxonomic units (OTUs) based on 97% sequence identity, and compared (blastn) against the Greengenes database for assigning taxonomic affiliation [66].

### 16S rRNA phylogenetic reconstruction

The OTUs comprising >0.5% of the total bacterial community in either of the termite hindgut paunch microbiota were identified as major OTUs. The representative (centroid) sequences for these OTUs were used to reconstruct a phylogenetic tree, using the quality-trimmed 200 bp from the V8 region amplified from the pyrotag analysis. Sequences were aligned using the SINA Webaligner (http://www.arb-silva.de/aligner/) and imported into the SILVA database (release 102) [67] using the ARB software package [68]. A backbone phylogenetic tree was constructed with over 1,000 full-length reference sequences using FastTree [69]. Short pyrotag reads were added to this tree according to maximum-parsimony criteria as provided by ARB. Finally, all reference sequences were removed without altering the calculated tree topology.

### Metagenome sequencing, assembly and annotation

Sanger sequencing from a small insert (3 kb) library and 454 GS FLX Titanium sequencing were performed according to DOE Joint Genome Institute standard operating procedures for shotgun sequencing (http://www.jgi.doe.gov/sequencing/protocols/index.html). The 454 raw reads were submitted to the NCBI Short Read Archive (SRA) with Submission IDs of SRA057543 and SRA057337 for *A. wheeleri* and *N. corniger* metagenomes, respectively. Sequencing reads were quality trimmed using LUCY [70]. The quality trimmed Sanger and 454 reads were then combined and assembled using the Newbler assembler software (version 2.0, 454 Life Sciences), with a minimum overlap length (ml) of 30 and a minimum overlap identity (mi) of 0.95. The assemblies (including assembled contigs and unassembled singlets) were uploaded to the Integrated Microbial Genomes with Microbiome (IMG/M) system (img.jgi.doe.gov/m) for gene calling with IMG/M Metagenome Gene Calling method and function annotation [71]. The annotated *A. wheeleri* and *N. corniger* metagenomes are publicly available with IMG/M Taxon Object IDs of 2030936000 and 2030936001 respectively. The average read depth for each contig was also uploaded to IMG/M as an estimator of the abundance of a population, from which the sequence was derived. The summary statistics for sequencing, assembly and annotation are shown in **Table S1**.

### Metagenome comparison and statistical analysis

Gene-centric analysis was conducted to reveal the system commonalities and differences in function. We compared metagenomes based on the functional units of pfam, COG, COG pathways or functional categories. Results based on pfams were largely consistent with those derived from COGs. Therefore, the results were mostly interpreted based on COGs, and in some cases, results from pfams were also listed if corresponding COGs were not available (**Table S4**). In addition, a Costa Rican wood-feeding *Nasutitermes* metagenome [6] was included as an additional control to verify the differences observed between the cow dung-feeding *A. wheeleri* and the laboratory wood-feeding *N. corniger*. The abundance of each functional unit is the total count of genes belonging to that unit, adjusted (multiplied) by individual population abundance, which was estimated using the average read depth of the corresponding contig. Despite the difference in community composition, the average genome size was comparable between the two metagenomes (**Table S7**). Therefore, the genome size-associated bias should be minimal, and thus the abundance of individual functional units was compared based on the odds ratio between metagenomes. By the IMG/M abundance profile tool, Z-LOR is defined as the natural logarithm of odds ratio (LOR) divided by the standard error of LOR. Z-LOR was used to indicate the strength of difference between metagenomes, and P-values were calculated from Z-LOR, and adjusted for multiple hypothesis testing with a false discovery rate cutoff of 0.05 to identify statistically significant differences.

### RNA extraction and cDNA Illumina library construction

Total RNA was extracted from the P3 segments pooled from ∼30 worker termites using the RiboPure™ Bacteria Kit (Ambion, Austin, TX) with DNase I digestion, following the manufacturer's instructions. The mRNA was enriched by using MICROBExpress™ Bacterial mRNA Enrichment Kit (Ambion). A chemical fragmentation step was performed by incubating the mRNA-enriched RNAs in 1× fragmentation solution (Ambion) at 70°C for 5 min to generate average fragment sizes of ∼175–200 bp. The fragmented RNAs were then used to synthesize double-stranded cDNAs using the SuperScript II Double-Stranded cDNA Synthesis Kit (Invitrogen, Carlsbad, CA), with random hexamers for the first strand synthesis and nick translation for the second strand synthesis. Illumina libraries were prepared by using the Illumina genomic sample prep kit (Illumina, San Diego, CA), according to manufacturer's instructions. A gel electrophoresis purification step was performed to select ligation products ranging from 200 to 500 bp, using the MiniElute Gel Extraction Kit (Qiagen, Valencia, CA). A PCR with 15 cycles using adaptor primers was performed to enrich adaptor-modified cDNA fragments. For each library, one lane of single-end 34 bp and one lane of paired-end 76 bp reads were sequenced on the Illumina Genome Analyzer (GA) II platform. In addition, one lane of paired-end 113 bp reads was generated on the Illumina GAIIx platform for *N. corniger* and a total of nine lanes (including failed lanes with low qualities) were generated for *A. wheeleri* microbiota due to its higher diversity. In total there were 11.4 Gbp of raw sequences generated for *N. corniger* and 62.4 Gbp for *A. wheeleri* (**Table S3**).

### Metatranscriptomics bioinformatics

Due to the high microbial diversity of our samples, each metagenome (MG) was combined with its corresponding *de novo* assembled metatranscriptome (MT) to generate a more complete reference (MG+MT) for mapping cDNA reads for gene expression. **Figure S4** summarized the analysis workflow. Briefly, to generate the metagenome-guided *de novo* metatranscriptome assembly (MT), a rapid rRNA filter using DUK, an efficient Kmer matching tool developed by JGI [72], was first applied to remove a large fraction of rRNA reads. Adapter and low quality sequences were trimmed using in-house tools and the overlapping paired reads were individually assembled using Phrap (http://www.phrap.org, version 1.0.1). The assembled mate pairs and the remaining reads were then used for a *de novo* assembly with Velvet [73] to generate the initial metatranscriptome contigs, which were uploaded to IMG/M for annotation (Taxon Object ID is 2162886001 and 2162886000 for the *de novo* assembled *A. wheeleri* and *N. corniger* metatranscriptome respectively). These contigs and the predicted genes from corresponding metagenome were coassembled using Newbler (454 Life Sciences). The transcriptome reads were then aligned back to these contigs using BWA (version 1.2.2) [74] and the paired-read alignments were used to associate the reads into scaffold-groups. Each scaffold's reads were either iteratively assembled with Velvet using multiple, increasing kmers, or assembled with Newbler if the estimated read depth was 200 or less. This allowed each transcriptional unit to be assembled individually with accurate estimates of read coverage and insert size distribution and each assembly was sufficiently small to be performed on commodity hardware on the compute-cluster. These final metatranscriptome contigs (MT) were used for functional annotation by the IMG/M pipeline. Later analysis indicated that low-coverage transcripts were not adequately assembled into single contigs with this method, so the transcriptome contigs were assembled with the predicted genes from the metagenome assembly using Phrap to generate a more complete reference (MG+MT), followed by IMG/M functional annotation. Annotated coassemblies are publicly available, with Taxon Object IDs of 2228664018 and 2228664019 for *A. wheeleri* and *N. corniger* respectively. For gene expression levels, the quality-trimmed cDNA reads were mapped to MG+MT genes using BWA. As a considerable amount of cDNA reads were found to derive from eukaryotes, especially from the hosts, proteins that shared >30% similarities to eukaryote sequences were excluded from analysis. Median read coverage over each gene was used to indicate its expression. Expression levels of genes from the same COG or pfam were summed to perform comparative analysis based on COGs or pfams, where the expression of each function was compared by using its odds ratio between the two metatranscriptomes. Similar to the metagenome comparison, Z-LOR was used to indicate the strength of difference between metatranscriptomes, and P-values were calculated from Z-LOR, and adjusted for multiple hypothesis testing with a false discovery rate cutoff of 0.05 to identify statistically significant differences.

## Supporting Information

Figure S1 Microbial community diversity and phylogenetic composition. (**a**) Rarefaction curves labeled with the Shannon diversity index (*H*). (**b**, **c**, **d**, **e**) Bacterial community composition at the phylum level by 16S rRNA pyrotag (**b**), by metagenome genes from 38 IMG/M phylogenetic marker COGs (**c**), and by all genes with function prediction in the metagenomes (**d**) and in the *de novo* assembled metatranscriptomes (**e**), respectively. In (**c**, **d**, **e**), sequence homology-based classification was performed by Blastp against NR, and taxonomy was assigned by using the lowest common ancestor algorithm of MEGAN. Taxonomic distribution was then weighted by read depths or expression levels of individual genes for metagenomes or metatranscriptomes respectively.
(PDF)

Figure S2 Hierarchical clustering of metagenomes based on similarities in pfam profiles. Proximity of grouping indicates the relative degree of similarity of samples to each other. The number of genes from each pfam was normalized by the total number of genes from all pfams found in each metagenome to generate the pfam profile. The number in the parenthesis is the metagenome Taxon Object ID in IMG/M.
(PDF)

Figure S3 Natural logarithm of odds ratios of COG categories when comparing the three termite hindgut P3 metagenomes to the average of a total of 149 metagenomes not associated with termites (**a**) and to the average of all 2456 bacterial genomes (**b**) in the IMG/M database as publically available in March, 2011.
(PDF)

Figure S4 Bioinformatic workflow of metatranscriptome analyses.
(PDF)

Figure S5 Transcript distribution among COG categories.
(PDF)

Figure S6 A cluster heatmap showing the clustering patterns of lignocellulose-degrading communities based on GH composition (the relative abundance of individual GHs in total GHs, based on gene counts). Only the GHs with the relative abundance ≥2% in any one of these microbiomes were shown in this heatmap.
(PDF)

Figure S7 Relative abundance of each GH in the four categories of GHs listed above for metagenomes (**a**) and metatranscriptomes (**b**).
(PDF)

Figure S8 Taxonomic distribution of methyl-accepting chemotaxis protein (MCP) family by MEGAN using Blastp results against the NR database.
(PDF)

Table S1 Metagenome sequencing, assembly and annotation summary
(PDF)

Table S2 Phylogenetic marker COGs used in phylogenetic distribution
(PDF)

Table S3 Metatranscriptome sequencing, assembly, annotation and read mapping summary
(PDF)

Table S4 Major functions differentially represented between *A. wheeleri* and *Nasutitermes* spp. Metagenomes
(PDF)

Table S5 Major functions differentially expressed between *A. wheeleri* and *N. corniger* metatranscriptomes
(PDF)

Table S6 Glycoside hydrolase (GH) abundance in metagenomes
(PDF)

Table S7 Estimated average genome size in metagenomes using single-copy phylogenetic markers
(PDF)

## References

[1] Brune A, Ohkuma M (2011) Role of the Termite Gut Microbiota in Symbiotic Digestion. In: Bignell DE, Roisin Y, Lo N, editors. Biology of Termites: a Modern Synthesis. Dordrecht: Springer pp. 439–475.

[2] Brune A, Emerson D, Breznak JA (1995) The Termite Gut Microflora as an Oxygen Sink: Microelectrode Determination of Oxygen and pH Gradients in Guts of Lower and Higher Termites. Appl Environ Microbiol 61: 2681–2687. 16535076 10.1128/aem.61.7.2681-2687.1995 PMC1388494

[3] Brune A, Kühl M (1996) pH profiles of the extremely alkaline hindguts of soil-feeding termites (Isoptera: Termitidae) determined with microelectrodes. Journal of Insect Physiology 42: 1121.

[4] Schmitt-Wagner D, Brune A (1999) Hydrogen profiles and localization of methanogenic activities in the highly compartmentalized hindgut of soil-feeding higher termites (Cubitermes spp.). Applied And Environmental Microbiology 65: 4490–4496. 10508080 10.1128/aem.65.10.4490-4496.1999 PMC91598

[5] Köhler T, Dietrich C, Scheffrahn RH, Brune A (2012) High-Resolution Analysis of Gut Environment and Bacterial Microbiota Reveals Functional Compartmentation of the Gut in Wood-Feeding Higher Termites (Nasutitermes spp.). Applied and Environmental Microbiology 78: 4691–4701. 22544239 10.1128/AEM.00683-12 PMC3370480

[6] Warnecke F, Luginbuhl P, Ivanova N, Ghassemian M, Richardson TH, et al (2007) Metagenomic and functional analysis of hindgut microbiota of a wood-feeding higher termite. Nature 450: 560–565. 18033299 10.1038/nature06269

[7] Ferrar P, Watson JAL (1970) Termites (Isoptera) Association with dung in Australia. Australian Journal of Entomology 9: 100–102.

[8] Wood TG (1978) Food and feeding habits of termites. In: Brian MV, editor. Production Ecology of Ants and Termites Cambridge: University Press. pp. 55–80.

[9] Freymann BP, Buitenwerf R, Desouza O, Olff H (2008) The importance of termites (Isoptera) for the recycling of herbivore dung in tropical ecosystems: a review. European Journal Of Entomology 105: 165–173.

[10] Ley RE, Hamady M, Lozupone C, Turnbaugh PJ, Ramey RR, et al (2008) Evolution of mammals and their gut microbes. Science 320: 1647–1651. 18497261 10.1126/science.1155725 PMC2649005

[11] Ettershank G, Ettershank JA, Whitford WG (1980) Location of food sources by subterranean termites. Environmental Entomology 9: 645–648.

[12] Scheffrahn RH, Krecek J, Szalanski AL, Austin JW (2005) Synonymy of neotropical arboreal termites Nasutitermes corniger and N. costalis (Isoptera : Termitidae : Nasutitermitinae), with evidence from morphology, genetics, and biogeography. Annals Of The Entomological Society Of America 98: 273–281.

[13] Hongoh Y, Deevong P, Inoue T, Moriya S, Trakulnaleamsai S, et al (2005) Intra- and Interspecific Comparisons of Bacterial Diversity and Community Structure Support Coevolution of Gut Microbiota and Termite Host. Appl Environ Microbiol 71: 6590–6599. 16269686 10.1128/AEM.71.11.6590-6599.2005 PMC1287746

[14] Hongoh Y, Deevong P, Hattori S, Inoue T, Noda S, et al (2006) Phylogenetic diversity, localization, and cell morphologies of members of the candidate phylum TG3 and a subphylum in the phylum Fibrobacteres, recently discovered bacterial groups dominant in termite guts. Applied And Environmental Microbiology 72: 6780–6788. 17021231 10.1128/AEM.00891-06 PMC1610327

[15] Brune A (2006) Symbiotic Associations between Termites and Prokaryotes. In: Dworkin M, Falkow S, Rosenberg E, Schleifer K-H, Stackebrandt E, editors. The Prokaryotes. New York: Springer. pp. 439–474.

[16] Hongoh Y, Ohkuma M, Kudo T (2003) Molecular analysis of bacterial microbiota in the gut of the termite Reticulitermes speratus (Isoptera; Rhinotermitidae). Fems Microbiology Ecology 44: 231–242. 19719640 10.1016/S0168-6496(03)00026-6

[17] Schmitt-Wagner D, Friedrich MW, Wagner B, Brune A (2003) Phylogenetic diversity, abundance, and axial distribution of bacteria in the intestinal tract of two soil-feeding termites (Cubitermes spp.). Applied And Environmental Microbiology 69: 6007–6017. 14532056 10.1128/AEM.69.10.6007-6017.2003 PMC201194

[18] Ballor NR, Leadbetter JR (2012) Patterns of [FeFe] hydrogenase diversity in the gut communities of lignocellulose-feeding higher termites. Applied and Environmental Microbiology: Published online ahead of print on 25 May 2012. 10.1128/AEM.08008-11 PMC3416405 22636002

[19] Yang H, Schmitt-Wagner D, Stingl U, Brune A (2005) Niche heterogeneity determines bacterial community structure in the termite gut (Reticulitermes santonensis). Environmental Microbiology 7: 916–932. 15946289 10.1111/j.1462-2920.2005.00760.x

[20] Shinzato N, Muramatsu M, Matsui T, Watanabe Y (2007) Phylogenetic analysis of the gut bacterial microflora of the fungus-growing termite Odontotermes formosanus. Biosci Biotechnol Biochem 71: 906–915. 17420599 10.1271/bbb.60540

[21] Tringe SG, von Mering C, Kobayashi A, Salamov AA, Chen K, et al (2005) Comparative Metagenomics of Microbial Communities. Science 308: 554–557. 15845853 10.1126/science.1107851

[22] Brune A, Friedrich M (2000) Microecology of the termite gut: structure and function on a microscale. Current Opinion in Microbiology 3: 263. 10851155 10.1016/s1369-5274(00)00087-4

[23] Breznak JA, Brune A (1994) Role of microorganisms in the digestion of lignocellulose by termites. Annu Rev Entomol 39: 453–487.

[24] Kumar P, Barrett DM, Delwiche MJ, Stroeve P (2009) Methods for Pretreatment of Lignocellulosic Biomass for Efficient Hydrolysis and Biofuel Production. Ind Eng Chem Res 48: 3713.

[25] Schmitt-Wagner D, Friedrich MW, Wagner B, Brune A (2003) Axial dynamics, stability, and interspecies similarity of bacterial community structure in the highly compartmentalized gut of soil-feeding termites (Cubitermes spp.). Applied And Environmental Microbiology 69: 6018–6024. 14532057 10.1128/AEM.69.10.6018-6024.2003 PMC201195

[26] Köhler T, Stingl U, Meuser K, Brune A (2008) Novel lineages of Planctomycetes densely colonize the alkaline gut of soil-feeding termites (Cubitermes spp.). Environmental Microbiology 10: 1260. 18279348 10.1111/j.1462-2920.2007.01540.x

[27] Thidarat N, Thongaram T, Uengwetwanit T, Pongpattanakitshote S, Eurwilaichitr L (2012) Metagenomic analysis of novel lignocellulose-degrading enzymes from higher termite guts inhabiting microbes. J Microbiol Biotechnol 22: 462–469. 22534292 10.4014/jmb.1108.08037

[28] Allgaier M, Reddy A, Park JI, Ivanova N, D'haeseleer P, et al (2010) Targeted Discovery of Glycoside Hydrolases from a Switchgrass-Adapted Compost Community. PLoS ONE 5: e8812 doi:8810.1371/journal.pone.0008812. 20098679 10.1371/journal.pone.0008812 PMC2809096

[29] Pope PB, Denman SE, Jones M, Tringe SG, Barry K, et al (2010) Adaptation to herbivory by the Tammar wallaby includes bacterial and glycoside hydrolase profiles different from other herbivores. Proceedings of the National Academy of Sciences 107: 14793–14798. 10.1073/pnas.1005297107 PMC2930436 20668243

[30] Zhu L, Wu Q, Dai J, Zhang S, Wei F (2011) Evidence of cellulose metabolism by the giant panda gut microbiome. Proceedings Of The National Academy Of Sciences Of The United States Of America 108: 17714–17719. 22006317 10.1073/pnas.1017956108 PMC3203778

[31] Brulc JM, Antonopoulos DA, Miller MEB, Wilson MK, Yannarell AC, et al (2009) Gene-centric metagenomics of the fiber-adherent bovine rumen microbiome reveals forage specific glycoside hydrolases. Proceedings Of The National Academy Of Sciences Of The United States Of America 106: 1948–1953. 19181843 10.1073/pnas.0806191105 PMC2633212

[32] Hess M, Sczyrba A, Egan R, Kim T-W, Chokhawala H, et al (2011) Metagenomic Discovery of Biomass-Degrading Genes and Genomes from Cow Rumen. Science 331: 463–467. 21273488 10.1126/science.1200387

[33] Scheller HV, Ulvskov P (2010) Hemicelluloses. Annual Review of Plant Biology 61: 263–289. 10.1146/annurev-arplant-042809-112315 20192742

[34] Vogel J (2008) Unique aspects of the grass cell wall. Current Opinion in Plant Biology 11: 301–307. 18434239 10.1016/j.pbi.2008.03.002

[35] Greenham PM (1972) The Effects of the Variability of Cattle Dung on the Multiplication of the Bushfly (Musca vetustissima Walk). Journal of Animal Ecology 41: 153.

[36] Hoekstra NJ, Bosker T, Lantinga EA (2002) Effects of cattle dung from farms with different feeding strategies on germination and initial root growth of cress (Lepidium sativum L.). Agriculture, Ecosystems & Environment 93: 189.

[37] Saxena KK, Nath K, Srivastava SK (1989) The effect of using dung from cattle fed high-, low-or no-concentrate rations, on the quality and nutritive value of slurry from a biogas plant. Biological Wastes 28: 73–79.

[38] Scurfield G, Nicholls PW (1970) Amino-Acid Composition of Wood Proteins. J Exp Bot 21: 857–868.

[39] Nene YL. Utilizing traditional knowledge in agriculture. In: Balasubramanian AV, Nirmala-Devi TD, editors; 2006 3–5 July 2006; Bangalore. Centre for Indian Knowledge Systems. pp. 32–38.

[40] Ji R, Brune A (2001) Transformation and mineralization of C-14-labeled cellulose, peptidoglycan, and protein by the soil-feeding termite Cubitermes orthognathus. Biology And Fertility Of Soils 33: 166–174.

[41] Ji R, Brune A (2006) Nitrogen Mineralization, Ammonia Accumulation, and Emission of Gaseous NH3 by Soil-Feeding Termites. Biogeochemistry 78: 267.

[42] Subrahmanyam PVR, Sastry CA, Rao AVSP, Pillai SC (1960) Amino Acids in Sewage Sludges. Journal (Water Pollution Control Federation) 32: 344–350.

[43] Vartoukian SR, Palmer RM, Wade WG (2007) The division “Synergistes”. Anaerobe 13: 99–106. 17631395 10.1016/j.anaerobe.2007.05.004

[44] Hugenholtz P, Hooper SD, Kyrpides NC (2009) Focus: *Synergistetes* . Environmental Microbiology 11: 1327–1329. 19635113 10.1111/j.1462-2920.2009.01949.x

[45] Slaytor M, Chappell DJ (1994) Nitrogen metabolism in termites. Comparative Biochemistry and Physiology Part B: Comparative Biochemistry 107B: 1–10.

[46] Silberbacha M, Hüserb A, Kalinowskib J, Pühlerc A, Waltera B, et al (2005) DNA microarray analysis of the nitrogen starvation response of Corynebacterium glutamicum. Journal of Biotechnology 119: 357–367. 15935503 10.1016/j.jbiotec.2005.04.007

[47] Conroy MJ, Durand A, Lupo D, Li X-D, Bullough PA, et al (2007) The crystal structure of the Escherichia coli AmtB–GlnK complex reveals how GlnK regulates the ammonia channel. Proceedings of the National Academy of Sciences 104: 1213–1218. 10.1073/pnas.0610348104 PMC1783118 17220269

[48] Antikainen R, Haapanen R, Rekolainen S (2004) Flows of nitrogen and phosphorus in Finland - the forest industry and use of wood fuels. Journal of Cleaner Production 12: 919.

[49] McDowell RW, Stewart I (2005) Phosphorus in fresh and dry dung of grazing dairy cattle, deer, and sheep: Sequential fraction and phosphorus-31 nuclear magnetic resonance analyses. Journal Of Environmental Quality 34: 598–607. 15758113 10.2134/jeq2005.0598

[50] Pester M, Brune A (2007) Hydrogen is the central free intermediate during lignocellulose degradation by termite gut symbionts. ISME J 1: 551. 18043656 10.1038/ismej.2007.62

[51] Brauman A, Kane MD, Labat M, Breznak JA (1992) Genesis of acetate and methane by gut bacteria of nutritionally diverse termites. Science 257: 1384–1387. 17738281 10.1126/science.257.5075.1384

[52] Ohkuma M, Brune A (2011) Diversity, Structure, and Evolution of the Termite Gut Microbial Community. In: Bignell DE, Roisin Y, Lo N, editors. Biology of Termites: a Modern Synthesis: Springer. pp. 413–438.

[53] Brauman A, Dore J, Eggleton P, Bignell D, Breznak JA, et al (2001) Molecular phylogenetic profiling of prokaryotic communities in guts of termites with different feeding habits. Fems Microbiology Ecology 35: 27–36. 11248387 10.1111/j.1574-6941.2001.tb00785.x

[54] Matson EG, Zhang X, Leadbetter JR (2010) Selenium controls transcription of paralogous formate dehydrogenase genes in the termite gut acetogen, Treponema primitia. Environmental Entomology 12: 2245–2258. 10.1111/j.1462-2920.2010.02188.x 21966917

[55] Zhang X, Matson EG, Leadbetter JR (2011) Genes for selenium dependent and independent formate dehydrogenase in the gut microbial communities of three lower, wood-feeding termites and a wood-feeding roach. Environmental Entomology 13: 307–323. 10.1111/j.1462-2920.2010.02330.x 20819103

[56] Ballor NR, Leadbetter JR (2012) Analysis of Extensive [FeFe] Hydrogenase Gene Diversity Within the Gut Microbiota of Insects Representing Five Families of Dictyoptera. Microbial Ecology 63: 586–595. 21935609 10.1007/s00248-011-9941-5

[57] Ottesen EA, Leadbetter JR (2011) Formyltetrahydrofolate synthetase gene diversity in the guts of higher termites with different diets and lifestyles. Applied And Environmental Microbiology 77: 3461–3467. 21441328 10.1128/AEM.02657-10 PMC3126463

[58] Salmassi TM, Leadbetter JR (2003) Analysis of genes of tetrahydrofolate-dependent metabolism from cultivated spirochaetes and the gut community of the termite Zootermopsis angusticollis. Microbiology 149: 2529–2537. 12949177 10.1099/mic.0.26351-0

[59] Charon NW, Greenberg EP, Koopman MBH, Limberger RJ (1992) Spirochete chemotaxis, motility, and the structure of the spirochetal periplasmic flagella. Research in Microbiology 143: 597. 1475520 10.1016/0923-2508(92)90117-7

[60] Charon NW, Goldstein SF (2002) Genetics of motility and chemotaxis of a fascinating group of bacteria: The Spirochetes. Annual Review of Genetics 36: 47–73. 10.1146/annurev.genet.36.041602.134359 12429686

[61] Fraser CM, Casjens S, Huang WM, Sutton GG, Clayton R, et al (1997) Genomic sequence of a Lyme disease spirochaete, Borrelia burgdorferi. Nature 390: 580–586. 9403685 10.1038/37551

[62] Bellgard MI, Wanchanthuek P, La T, Ryan K, Moolhuijzen P, et al (2009) Genome Sequence of the Pathogenic Intestinal Spirochete Brachyspira hyodysenteriae Reveals Adaptations to Its Lifestyle in the Porcine Large Intestine. PLoS ONE 4: e4641. 19262690 10.1371/journal.pone.0004641 PMC2650404

[63] Russell JB, Mantovani HC (2002) The bacteriocins of ruminal bacteria and their potential as an alternative to antibiotics. Journal Of Molecular Microbiology And Biotechnology 4: 347–355. 12125815

[64] Constantino R (2002) The pest termites of South America: taxonomy, distribution and status. Journal of Applied Entomology 126: 355–365.

[65] Engelbrektson A, Kunin V, Wrighton KC, Zvenigorodsky N, Chen F, et al (2010) Experimental factors affecting PCR-based estimates of microbial species richness and evenness. ISME J 4: 642–647. 20090784 10.1038/ismej.2009.153

[66] Kunin V, Engelbrektson A, Ochman H, Hugenholtz P (2010) Wrinkles in the rare biosphere: pyrosequencing errors can lead to artificial inflation of diversity estimates. Environmental Microbiology 12: 118–123. 19725865 10.1111/j.1462-2920.2009.02051.x

[67] Pruesse E, Quast C, Knittel K, Fuchs BM, Ludwig W, et al (2007) SILVA: a comprehensive online resource for quality checked and aligned ribosomal RNA sequence data compatible with ARB. Nucleic Acids Research 35: 7188–7196. 17947321 10.1093/nar/gkm864 PMC2175337

[68] Ludwig W, Strunk O, Westram R, Richter L, Meier H, et al (2004) ARB: a software environment for sequence data. Nucleic Acids Research 32: 1363–1371. 14985472 10.1093/nar/gkh293 PMC390282

[69] Price MN, Dehal PS, Arkin AP (2009) FastTree: Computing Large Minimum Evolution Trees with Profiles instead of a Distance Matrix. Molecular Biology and Evolution 26: 1641–1650. 19377059 10.1093/molbev/msp077 PMC2693737

[70] Chou H-H, Holmes MH (2001) DNA sequence quality trimming and vector removal. Bioinformatics 17: 1093–1104. 11751217 10.1093/bioinformatics/17.12.1093

[71] Mavromatis K, Ivanova N, Chen A, Szeto E, Markowitz V, et al (2009) Standard Operating Procedure for the Annotations of Microbial Genomes by the Production Genomic Facility of the DOE JGI. Standards in Genomic Sciences 1 doi:10.4056/sigs.4632. 10.4056/sigs.632 PMC3035208 21304638

[72] Li M, Copeland A, Han J (2011) DUK - A Fast and Efficient Kmer Based Sequence Matching Tool. Lawrence Berkeley National Laboratory. LBNL Paper LBNL-4516E-Poster p.

[73] Zerbino DR, Birney E (2008) Velvet: Algorithms for de novo short read assembly using de Bruijn graphs. Genome Research 18: 821–829. 18349386 10.1101/gr.074492.107 PMC2336801

[74] Li H, Durbin R (2009) Fast and accurate short read alignment with Burrows-Wheeler transform. Bioinformatics 25: 1754–1760. 19451168 10.1093/bioinformatics/btp324 PMC2705234

