# Supplementary material for: Comparative Metagenomic and Metatranscriptomic Analysis of Hindgut Paunch Microbiota in Wood- and Dung-Feeding Higher Termites
Source: PLoS One. 2013 Apr 12;8(4):e61126. doi: 10.1371/journal.pone.0061126 (PMC3625147; doi:10.1371/journal.pone.0061126)
Supplement: Figure S1 — Microbial community diversity and phylogenetic composition. (a) Rarefaction curves labeled with the Shannon diversity index (H). (b, c, d, e) Bacterial community composition at the phylum level by 16S rRNA pyrotag (b), by metagenome genes from 38 IMG/M phylogenetic marker COGs (c), and by all genes with function prediction in the metagenomes (d) and in the de novo assembled metatranscriptomes (e), respectively. In (c, d, e), sequence homology-based classification was performed by Blastp against NR, and taxonomy was assigned by using the lowest common ancestor algorithm of MEGAN. Taxonomic distribution was then weighted by read depths or expression levels of individual genes for metagenomes or metatranscriptomes respectively. (PDF) [file pone.0061126.s001.pdf]

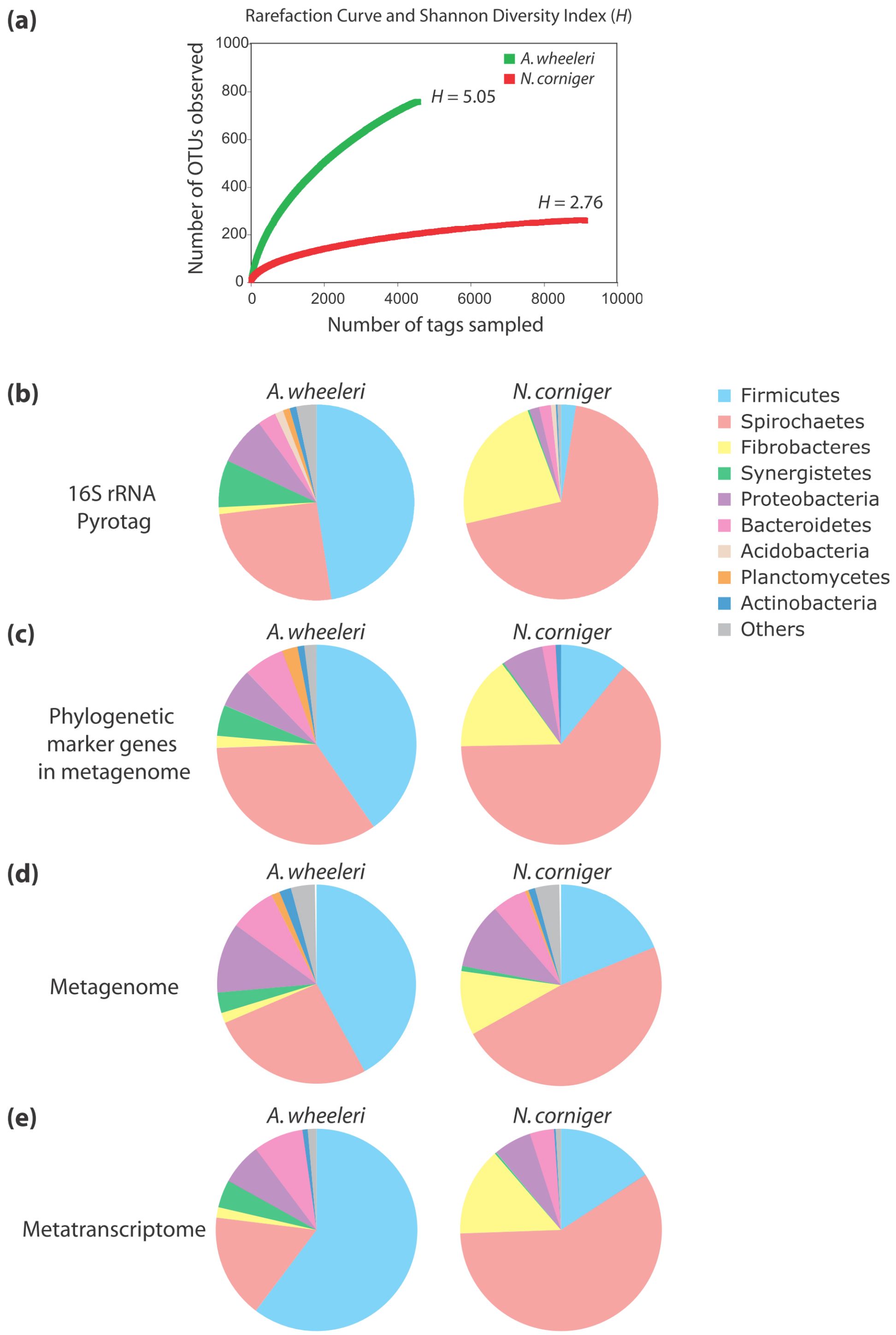

Figure S1. Microbial community diversity and phylogenetic composition. (**a**) Rarefaction curves labeled with the Shannon diversity index (*H*). (**b**, **c**, **d**, **e**) Bacterial community composition at the phylum level by 16S rRNA pyrotag (**b**), by metagenome genes from 38 IMG/M phylogenetic marker COGs (**c**), and by all genes with function prediction in the metagenomes (**d**) and in the *de novo* assembled metatranscriptomes (**e**), respectively. In (**c**, **d**, **e**), sequence homology-based classification was performed by Blastp against NR, and taxonomy was assigned by using the lowest common ancestor algorithm of MEGAN. Taxonomic distribution was then weighted by read depths or expression levels of individual genes for metagenomes or metatranscriptomes respectively.
