# Supplementary material for: Comparative Metagenomic and Metatranscriptomic Analysis of Hindgut Paunch Microbiota in Wood- and Dung-Feeding Higher Termites
Source: PLoS One. 2013 Apr 12;8(4):e61126. doi: 10.1371/journal.pone.0061126 (PMC3625147; doi:10.1371/journal.pone.0061126)
Supplement: Figure S2 — Hierarchical clustering of metagenomes based on similarities in pfam profiles. Proximity of grouping indicates the relative degree of similarity of samples to each other. The number of genes from each pfam was normalized by the total number of genes from all pfams found in each metagenome to generate the pfam profile. The number in the parenthesis is the metagenome Taxon Object ID in IMG/M. (PDF) [file pone.0061126.s002.pdf]

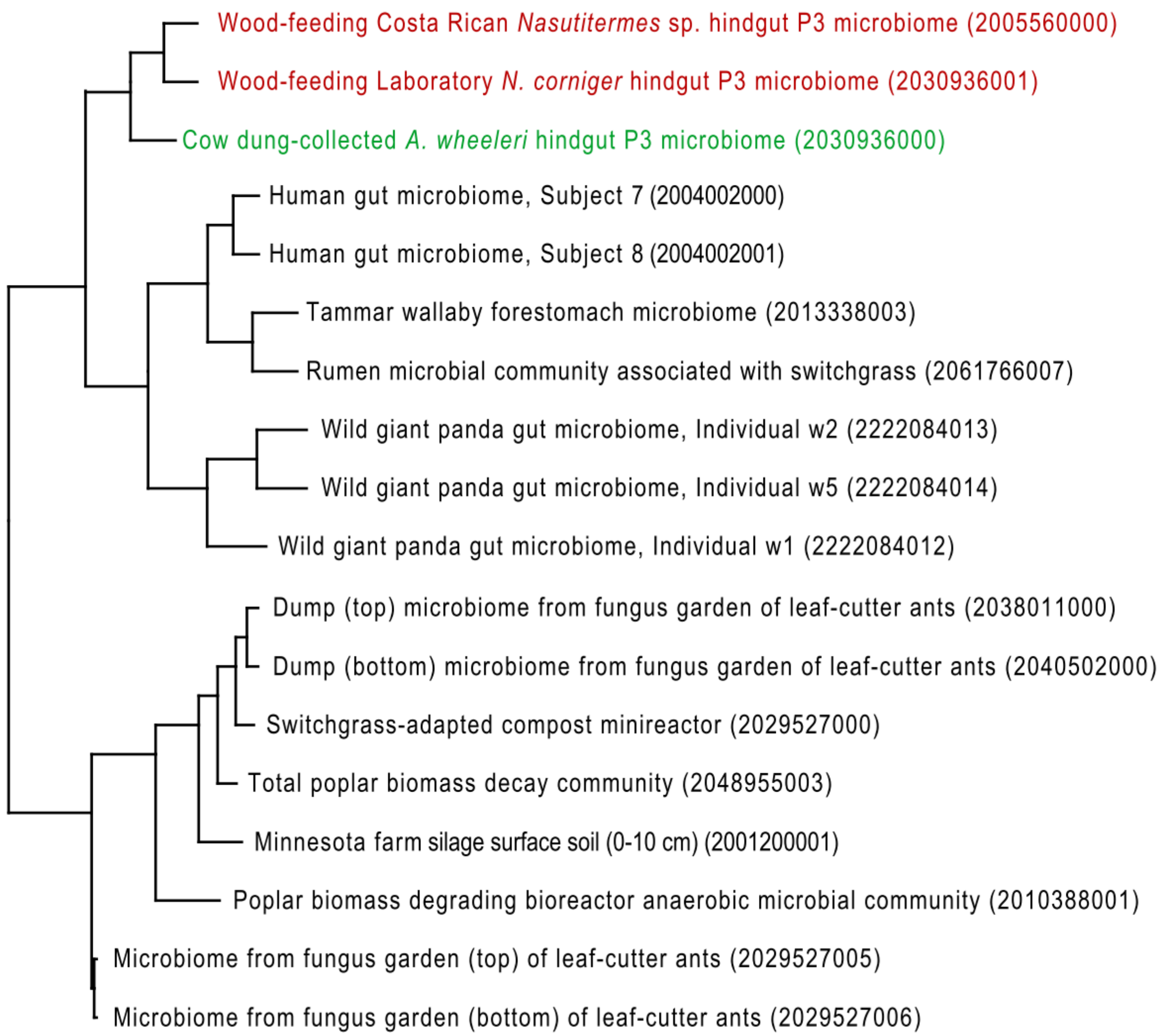

Figure S2. Hierarchical clustering of metagenomes based on similarities in pfam profiles. Proximity of grouping indicates the relative degree of similarity of samples to each other. The number of genes from each pfam was normalized by the total number of genes from all pfams found in each metagenome to generate the pfam profile. The number in the parenthesis is the metagenome Taxon Object ID in IMG/M.
