# Supplementary material for: Comparative Metagenomic and Metatranscriptomic Analysis of Hindgut Paunch Microbiota in Wood- and Dung-Feeding Higher Termites
Source: PLoS One. 2013 Apr 12;8(4):e61126. doi: 10.1371/journal.pone.0061126 (PMC3625147; doi:10.1371/journal.pone.0061126)
Supplement: Figure S3 — Natural logarithm of odds ratios of COG categories when comparing the three termite hindgut P3 metagenomes to the average of a total of 149 metagenomes not associated with termites (a) and to the average of all 2456 bacterial genomes (b) in the IMG/M database as publically available in March, 2011. (PDF) [file pone.0061126.s003.pdf]

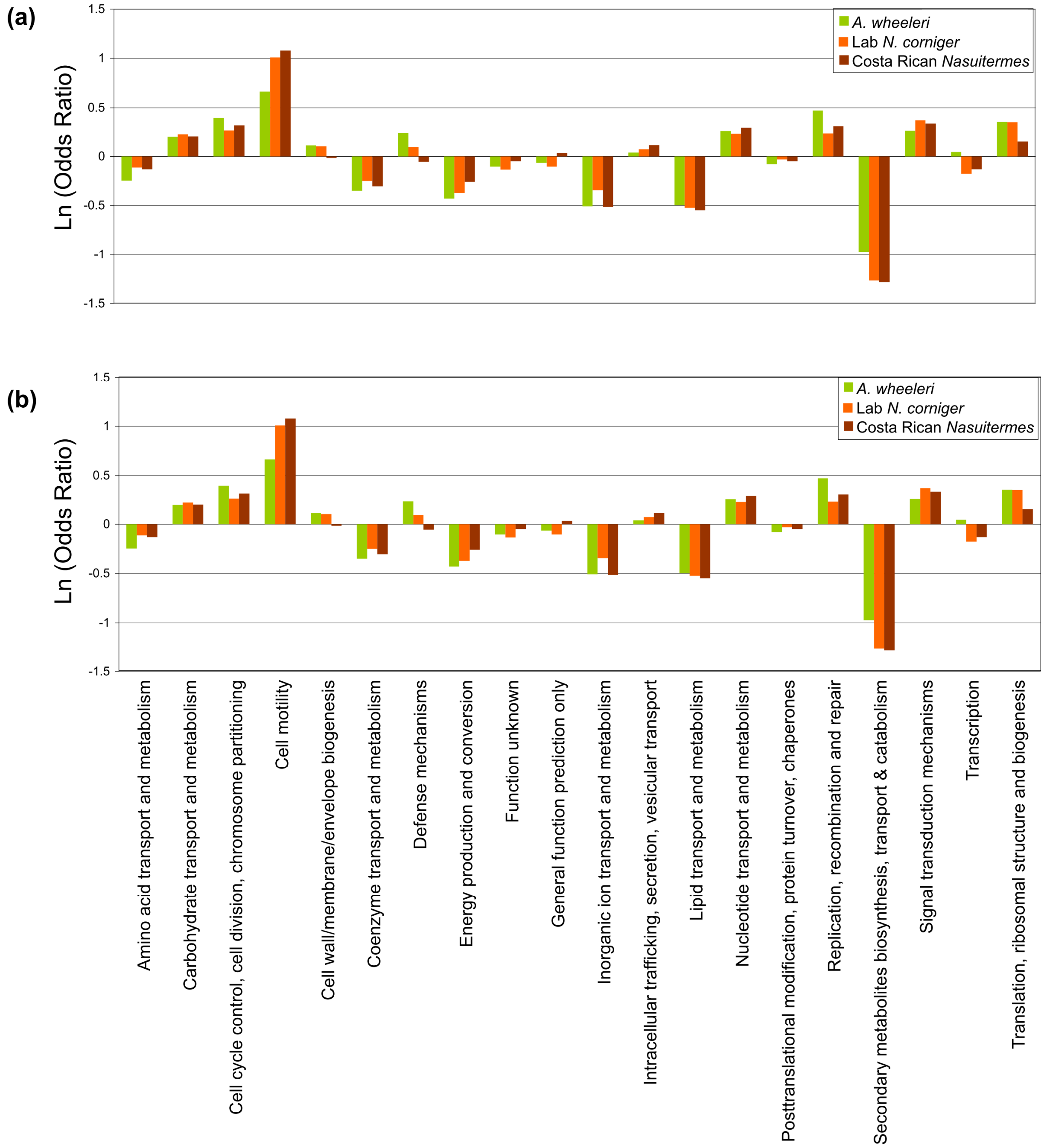

Figure S3. Natural logarithm of odds ratios of COG categories when comparing the three termite hindgut P3 metagenomes to the average of a total of 149 metagenomes not associated with termites (**a**) and to the average of all 2456 bacterial genomes (**b**) in the IMG/M database as publically available in March, 2011.
