# Supplementary figures and images for: Comparative Metagenomic and Metatranscriptomic Analysis of Hindgut Paunch Microbiota in Wood- and Dung-Feeding Higher Termites
Source: PLoS One. 2013 Apr 12;8(4):e61126. doi: 10.1371/journal.pone.0061126 (PMC3625147; doi:10.1371/journal.pone.0061126)

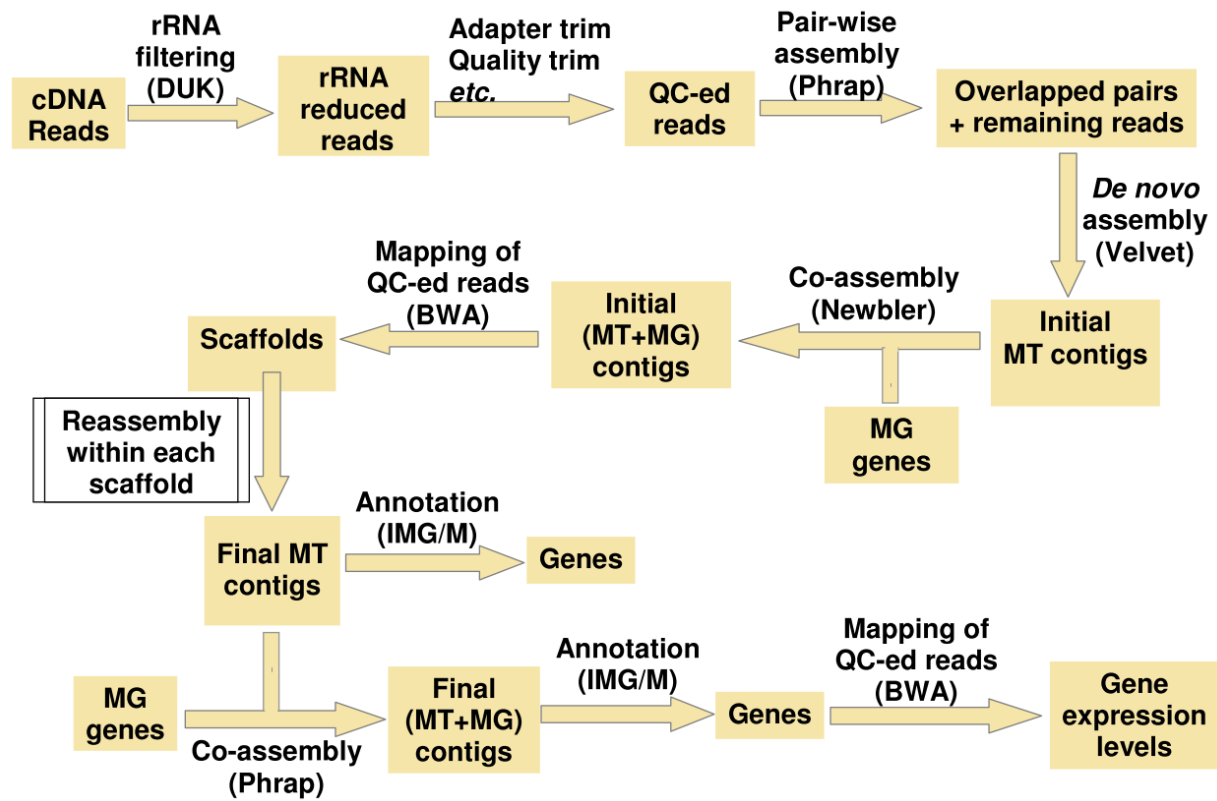

**Figure S4.** Bioinformatic workflow of metatranscriptome analyses.

Supplement: Figure S4 — Bioinformatic workflow of metatranscriptome analyses. (PDF) [file pone.0061126.s004.pdf]

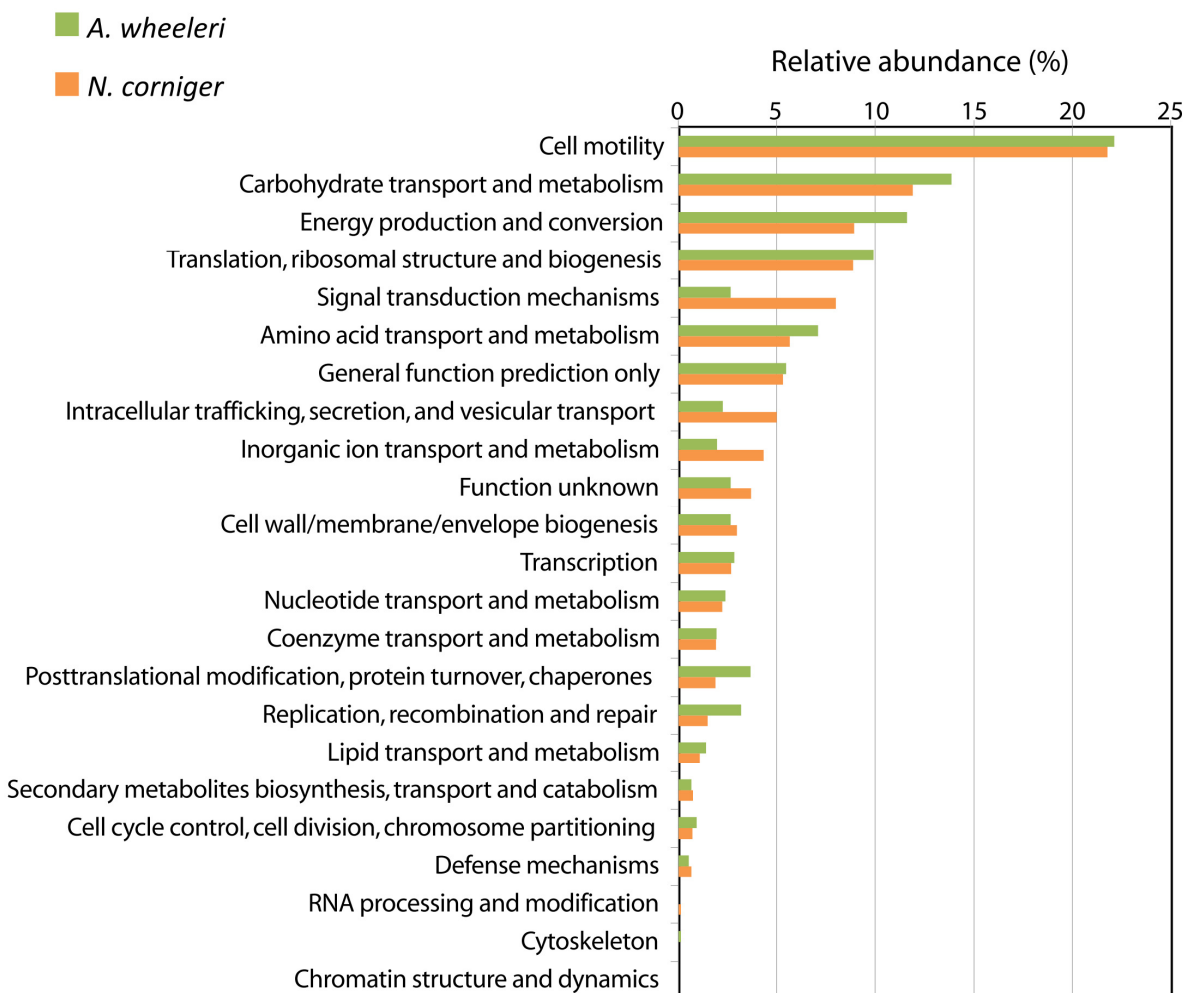

**Figure S5.** Transcript distribution among COG categories.

Supplement: Figure S5 — Transcript distribution among COG categories. (PDF) [file pone.0061126.s005.pdf]
