# Supplementary material for: Comparative Metagenomic and Metatranscriptomic Analysis of Hindgut Paunch Microbiota in Wood- and Dung-Feeding Higher Termites
Source: PLoS One. 2013 Apr 12;8(4):e61126. doi: 10.1371/journal.pone.0061126 (PMC3625147; doi:10.1371/journal.pone.0061126)
Supplement: Figure S6 — A cluster heatmap showing the clustering patterns of lignocellulose-degrading communities based on GH composition (the relative abundance of individual GHs in total GHs, based on gene counts). Only the GHs with the relative abundance ≥2% in any one of these microbiomes were shown in this heatmap. (PDF) [file pone.0061126.s006.pdf]

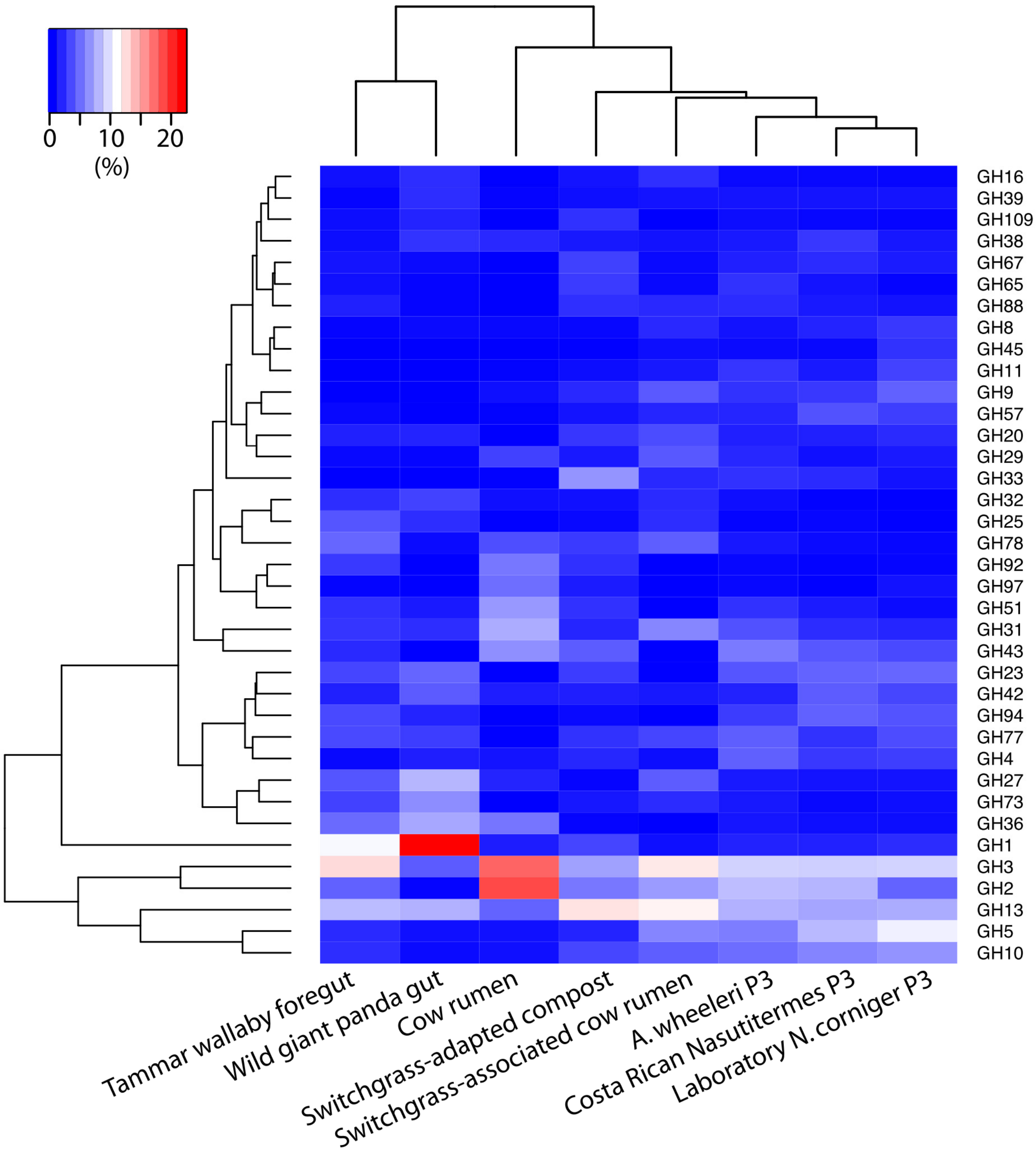

Figure S6. A cluster heatmap showing the clustering patterns of lignocellulose-degrading communities based on GH composition (the relative abundance of individual GHs in total GHs, based on gene counts). Only the GHs with the relative abundance ≥2% in any one of these microbiomes were shown in this heatmap.
