# Supplementary material for: Comparative Metagenomic and Metatranscriptomic Analysis of Hindgut Paunch Microbiota in Wood- and Dung-Feeding Higher Termites
Source: PLoS One. 2013 Apr 12;8(4):e61126. doi: 10.1371/journal.pone.0061126 (PMC3625147; doi:10.1371/journal.pone.0061126)
Supplement: Figure S7 — Relative abundance of each GH in the four categories of GHs listed above for metagenomes (a) and metatranscriptomes (b). (PDF) [file pone.0061126.s007.pdf]

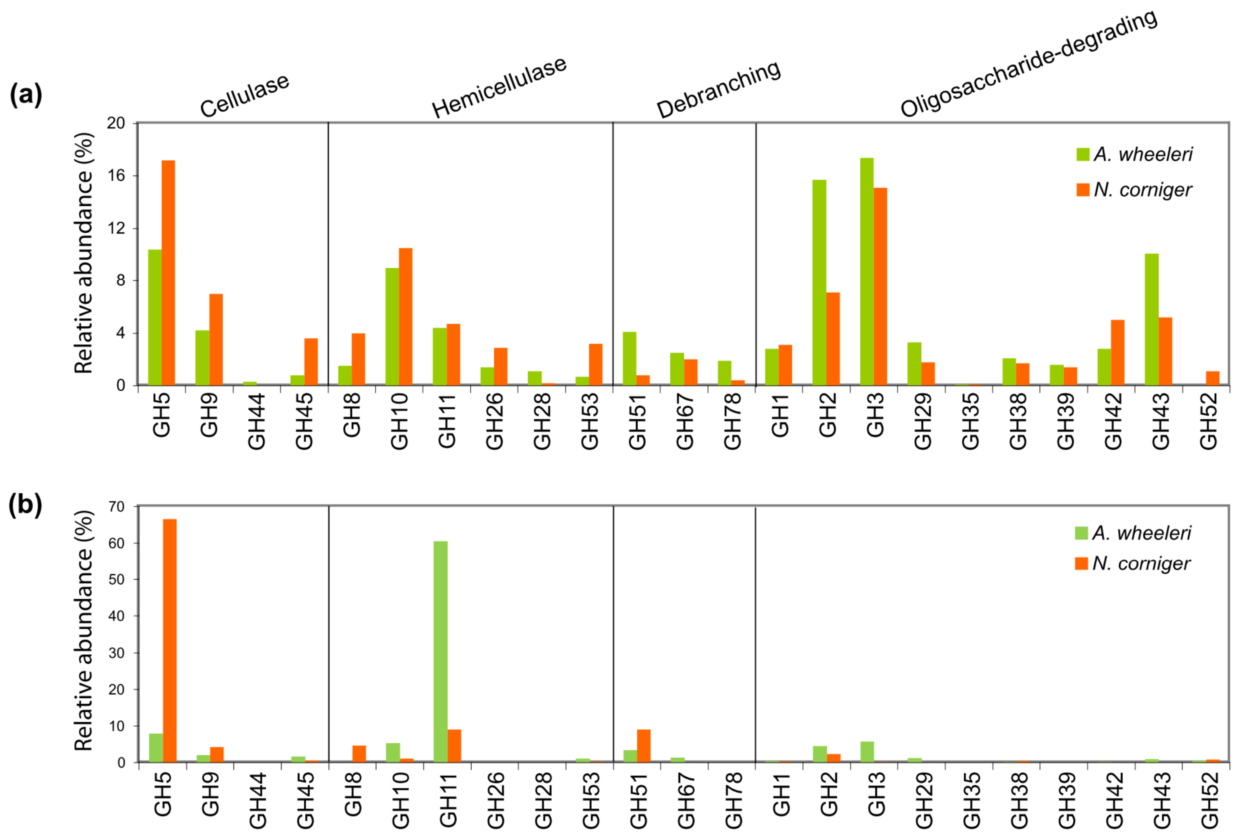

Figure S7. Relative abundance of each GH in the four categories of GHs listed above for metagenomes (**a**) and metatranscriptomes (**b**).
