# Supplementary material for: Comparative Metagenomic and Metatranscriptomic Analysis of Hindgut Paunch Microbiota in Wood- and Dung-Feeding Higher Termites
Source: PLoS One. 2013 Apr 12;8(4):e61126. doi: 10.1371/journal.pone.0061126 (PMC3625147; doi:10.1371/journal.pone.0061126)
Supplement: Figure S8 — Taxonomic distribution of methyl-accepting chemotaxis protein (MCP) family by MEGAN using Blastp results against the NR database. (PDF) [file pone.0061126.s008.pdf]

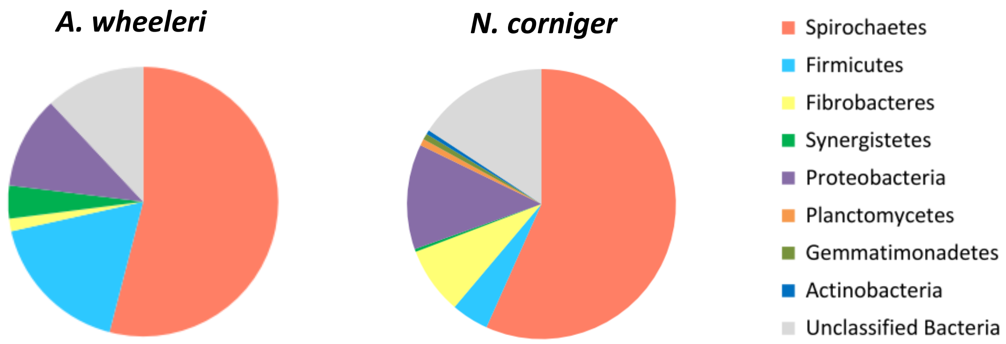

Figure S8. Taxonomic distribution of methyl-accepting chemotaxis protein (MCP) family by MEGAN using Blastp results against the NR database.
