# Supplementary material for: Comparative Metagenomic and Metatranscriptomic Analysis of Hindgut Paunch Microbiota in Wood- and Dung-Feeding Higher Termites
Source: PLoS One. 2013 Apr 12;8(4):e61126. doi: 10.1371/journal.pone.0061126 (PMC3625147; doi:10.1371/journal.pone.0061126)
Supplement: Table S1 — Metagenome sequencing, assembly and annotation summary (PDF) [file pone.0061126.s009.pdf]

Table S1. Metagenome sequencing, assembly and annotation summary

Metagenome	Sequencing			Assembly								Annotation				
	No. of Sanger reads	No. of 454 reads	Total bases used for assembly after read QC (bp)	Total bases went into contigs (bp)	Total length of contigs (bp)	Count of all contig	Count of large contig[a]	N50 of large contig	Mean of large contig	Largest contig (bp)	Count of singleton	No. of coding bases	No. of protein coding genes	Percent with function prediction (%)	Percent with COG (%)	Percent with pfam (%)
N. corniger (Lab colony)	8013	516,318	141,210,426	79,882,995	27,355,775	49,107	19,804	983	939	9833	193,472	44,733,336	150,782	48.2%	38.3%	39.7%
A. wheeleri (Cow dung)	12272	570,328	256,183,883	147,719,803	51,571,946	96,519	45,961	570	684	9229	213,123	100,984,684	321,461	44.2%	36.2%	37.7%

[a]Large contig is contig longer than 500 bp.
