# Supplementary material for: Comparative Metagenomic and Metatranscriptomic Analysis of Hindgut Paunch Microbiota in Wood- and Dung-Feeding Higher Termites
Source: PLoS One. 2013 Apr 12;8(4):e61126. doi: 10.1371/journal.pone.0061126 (PMC3625147; doi:10.1371/journal.pone.0061126)
Supplement: Table S2 — Phylogenetic marker COGs used in phylogenetic distribution (PDF) [file pone.0061126.s010.pdf]

Table S2. Phylogenetic marker COGs used in phylogenetic distribution

COG ID	Function Name	Single Copy[a]
COG0051	Ribosomal protein S10	Yes
COG0211	Ribosomal protein L27	Yes
COG0089	Ribosomal protein L23	Yes
COG0222	Ribosomal protein L7/L12	Yes
COG0256	Ribosomal protein L18	Yes
COG0093	Ribosomal protein L14	Yes
COG0233	Ribosome recycling factor	Almost
COG0691	tmRNA-binding protein	Yes
COG0197	Ribosomal protein L16/L10E	Yes
COG0200	Ribosomal protein L15	Yes
COG0094	Ribosomal protein L5	Yes
COG0100	Ribosomal protein S11	Yes
COG0292	Ribosomal protein L20	Yes
COG0099	Ribosomal protein S13	Yes
COG0669	Phosphopantetheine adenylyltransferase	Almost
COG0048	Ribosomal protein S12	Yes
COG0096	Ribosomal protein S8	Yes
COG0102	Ribosomal protein L13	Yes
COG0081	Ribosomal protein L1	Yes
COG0092	Ribosomal protein S3	Yes
COG0098	Ribosomal protein S5	Yes
COG0049	Ribosomal protein S7	Yes
COG0080	Ribosomal protein L11	Almost
COG0087	Ribosomal protein L3	Yes
COG0090	Ribosomal protein L2	Yes
COG0097	Ribosomal protein L6P/L9E	Yes
COG0088	Ribosomal protein L4	Yes
COG2255	Holliday junction resolvasome, helicase subunit	Almost
COG0150	Phosphoribosylaminoimidazole (AIR) synthetase	Yes
COG0541	Signal recognition particle GTPase	Yes
COG0632	Holliday junction resolvasome, DNA-binding subunit	Yes
COG0504	CTP synthase (UTP-ammonia lyase)	Yes
COG0216	Protein chain release factor A	Yes
COG0173	Aspartyl-tRNA synthetase	Almost
COG0481	Membrane GTPase LepA	Almost
COG0016	Phenylalanyl-tRNA synthetase alpha subunit	Yes
COG0556	Helicase subunit of the DNA excision repair complex	Yes
COG0533	Metal-dependent proteases with possible chaperone activity	Almost

[a] “Yes” means that genes belonging to this COG are single copy per genome. “Almost” means that genes belonging to this COG are single copy in most genomes.
