# Supplementary material for: Comparative Metagenomic and Metatranscriptomic Analysis of Hindgut Paunch Microbiota in Wood- and Dung-Feeding Higher Termites
Source: PLoS One. 2013 Apr 12;8(4):e61126. doi: 10.1371/journal.pone.0061126 (PMC3625147; doi:10.1371/journal.pone.0061126)
Supplement: Table S3 — Metatranscriptome sequencing, assembly, annotation and read mapping summary (PDF) [file pone.0061126.s011.pdf]

Table S3. Metatranscriptome sequencing, assembly, annotation and read mapping summary

		N. corniger	*A. wheeleri*
	Total bases in raw sequences generated (Mbp)	11,391	62,396
	No. of raw reads	123,229,584	443,635,388
	No. of reads after a fast rRNA filter	18,003,327	70,634,785
	No. of reads after QC	17,210,051	65,707,417
De novo assembly of metatranscriptome[a]	No. of contigs	11,048	17,546
	Min. contig length (bp)	100	100
	Max. contig length (bp)	1,944	2,946
	Average contig length (bp)	217	226
	Median contig length (bp)	150	155
	Total base in all contigs (bp)	2,392,193	3,960,696
Annotation of *De novo* assembled metatranscriptome	Total base in the annotated metatranscriptome (bp)	2,367,344	3,936,836
	No. of coding bases (bp)	1,568,780	3,011,488
	No. of contigs in annotated metatranscriptome	10,830	17,366
	No. of all predicted genes	6,470	13,514
	No. of rRNA genes	3,339	9,029
	No. of protein-coding genes	3,131	4,459
	No. of protein-coding genes with function prediction	1,438	1,581
	No. of genes with COG	1,229	1,160
	No. of genes with pfam	1,194	1,381
Reads mapped to the co-assembly of metagenome and metatranscriptome[b]	No. of reads mapped to the co-assembly	5,047,055	4,798,261
	No. of reads mapped to rRNA	3,604,184	3,457,426
	No. of reads mapped to non-rRNA	1,442,871	1,340,835
	No. of reads mapped to genes with COG	487,628	469,315
	No. of reads mapped to genes with pfam	538,298	503,242

[a]Contigs shorter than 100 bp were excluded as they are not reliable for functional annotation.

[b]Before the co-assembly, the large number of rRNA genes obtained through the *de novo* metatranscriptome assembly were excluded to focus on the assembly of functional genes. The number of reads mapped to rRNA would otherwise be much higher than the listed, if these rRNA genes had been included.
