# Supplementary material for: Comparative Metagenomic and Metatranscriptomic Analysis of Hindgut Paunch Microbiota in Wood- and Dung-Feeding Higher Termites
Source: PLoS One. 2013 Apr 12;8(4):e61126. doi: 10.1371/journal.pone.0061126 (PMC3625147; doi:10.1371/journal.pone.0061126)
Supplement: Table S4 — Major functions differentially represented between A. wheeleri and Nasutitermes spp. Metagenomes (PDF) [file pone.0061126.s012.pdf]

Table S4. Major functions differentially represented between *A. wheeleri* and *Nasutitermes* spp. metagenomes

Function ID	Function description	Estimated gene copies[a]			*A. wheeleri* vs. laboratory *N. corniger*			*Amitermes* vs. Costa Rican *Nasutitermes* sp.		
		Ami[b]	Lab Nas[b]	CR Nas[b]	Z-LOR[c]	p-value	Signifi-cant[d]	Z-LOR[c]	p-value	Signifi-cant[d]
**Glycoside hydrolases in plant cell wall degradation**										
pfam02449	Glyco_hydro_42: Oligosaccharide degrading	41	57	41	-4.6687	3E-06	Yes	-5.02625	5E-07	Yes
pfam08532	Glyco_hydro_42M: Oligosaccharide degrading	22	35	29	-4.00926	6.1E-05	Yes	-4.90337	9.4E-07	Yes
pfam00933	Glyco_hydro_3: Oligosaccharide degrading	268	205	120	-3.86579	0.00011	Yes	-2.79182	0.00524	Yes
pfam03512	Glyco_hydro_52: Oligosaccharide degrading	0	22	4	-3.6359	0.00028	Yes	-2.23273	0.02557	No
pfam00232	Glyco_hydro_1: Oligosaccharide degrading	70	62	30	-2.89659	0.00377	Yes	-1.2038	0.22867	No
pfam10566	Glyco_hydro_97: Oligosaccharide degrading	7	14	1	-2.85069	0.00436	Yes	0.78198	0.43422	No
pfam02837	Glyco_hydro_2_N: Oligosaccharide degrading	142	48	70	2.74517	0.00605	Yes	-2.75784	0.00582	Yes
pfam01055	Glyco_hydro_31: Oligosaccharide degrading	167	52	40	3.40358	0.00067	Yes	1.81374	0.06972	No
pfam07745	Glyco_hydro_53: Hemicellulase	17	64	26	-7.15545	8.3E-13	Yes	-4.92124	8.6E-07	Yes
pfam01270	Glyco_hydro_8: Hemicellulase	37	80	32	-7.03073	2.1E-12	Yes	-3.99692	6.4E-05	Yes
pfam00331	Glyco_hydro_10: Hemicellulase	225	208	119	-5.6994	1.2E-08	Yes	-4.17515	3E-05	Yes
pfam02156	Glyco_hydro_26: Hemicellulase	34	58	24	-5.37367	7.7E-08	Yes	-2.85709	0.00428	Yes
pfam00457	Glyco_hydro_11: Hemicellulase	110	94	23	-3.3417	0.00083	Yes	1.9851	0.04713	No
pfam00150	Cellulase, GH5: Cellulase	258	341	167	-10.9823	4.6E-28	Yes	-6.80117	1E-11	Yes
pfam02015	Glyco_hydro_45: Cellulase	20	71	7	-7.48051	7.4E-14	Yes	-0.13691	0.8911	No
pfam00759	Glyco_hydro_9: Cellulase	104	138	51	-7.00507	2.5E-12	Yes	-2.32518	0.02006	No
pfam05592	Bac_rhamnosid: Debranching	48	8	9	3.05183	0.00227	Yes	1.55292	0.12044	No
COG3534	Alpha-L-arabinofuranosidase: Debranching	217	57	42	4.79881	1.6E-06	Yes	1.65457	0.09801	No
**Cohesin and dockerin**										
pfam09603	Fib_succ_major, corresponding to TIGR02145	255	2420	687	-43.9205	0	Yes	-28.7156	2E-181	Yes
pfam00404	Dockerin_1	29	0	0	2.69491	0.00704	Yes	2.2195	0.02645	No
pfam00963	Cohesin	30	0	0	2.72976	0.00634	Yes	2.25409	0.02419	No
**Nitrogen fixation**										
COG2710	Nitrogenase molybdenum-iron protein, alpha and beta chains	227	295	121	-10.0305	1.1E-23	Yes	-6.52729	6.7E-11	Yes
COG0347	Nitrogen regulatory protein PII	89	90	47	-4.24258	2.2E-05	Yes	-4.02142	5.8E-05	Yes
COG1348	Nitrogenase subunit NifH (ATPase)	61	55	17	-2.79347	0.00521	Yes	-0.31277	0.75445	No
	**Sum of all COGs involved[e]**	**578**	**574**	**243**	**-10.5756**	**3.9E-26**	**Yes**	**-6.72356**	**1.8E-11**	**Yes**
**Peptidoglycan degradation (by COGs)**										
COG5632	N-acetylmuramoyl-L-alanine amidase	53	9	4	3.19057	0.00142	Yes	2.35414	0.01857	No
COG0860	N-acetylmuramoyl-L-alanine amidase	212	72	27	3.35195	0.0008	Yes	3.41394	0.00064	Yes

Function ID	Function description	Estimated gene copies[a]			*A. wheeleri* vs. laboratory *N. corniger*			*Amitermes* vs. Costa Rican *Nasutitermes* sp.		
		Ami[b]	Lab Nas[b]	CR Nas[b]	Z-LOR[c]	p-value	Signifi-cant[d]	Z-LOR[c]	p-value	Signifi-cant[d]
COG1686	D-alanyl-D-alanine carboxypeptidase	209	66	30	3.75365	0.00017	Yes	2.96017	0.00307	Yes
COG2173	D-alanyl-D-alanine dipeptidase	61	5	1	4.03868	5.4E-05	Yes	2.72541	0.00642	Yes
	**Sum of all COGs involved**	**665**	**201**	**84**	**7.05765**	**1.7E-12**	**Yes**	**5.9588**	**2.5E-09**	**Yes**
**Peptidoglycan degradation (by pfams)**										
pfam02016	Peptidase_S66: Muramoyl-tetrapeptide carboxypeptidase	57	1	0	3.38727	0.00071	Yes	2.90792	0.00364	Yes
pfam01520	Amidase_3: N-acetylmuramoyl-L-alanine amidase	206	68	27	3.44737	0.00057	Yes	4.50693	6.6E-06	Yes
pfam00768	Peptidase_S11: D-alanyl-D-alanine carboxypeptidase	178	53	26	3.74023	0.00018	Yes	3.87718	0.00011	Yes
pfam01471	PG_binding_1: Putative peptidoglycan binding domain	85	1	8	3.79432	0.00015	Yes	3.38939	0.0007	Yes
pfam01510	Amidase_2: N-acetylmuramoyl-L-alanine amidase	91	18	9	3.85416	0.00012	Yes	3.4453	0.00057	Yes
pfam01427	Peptidase_M15: D-alanyl-D-alanine dipeptidase	57	5	1	3.87498	0.00011	Yes	2.90792	0.00364	Yes
	**Sum of all pfams involved**[e]	**723**	**165**	**78**	**8.09736**	**5.6E-16**	**Yes**	**8.09736**	**5.6E-16**	**Yes**
**Amino acid transport**										
COG3842	ABC-type spermidine/putrescine transport, ATPase components	191	161	69	-4.22695	2.4E-05	Yes	-2.45921	0.01392	No
COG4166	ABC-type oligopeptide transport, periplasmic component	145	128	40	-4.10925	4E-05	Yes	-0.4233	0.67208	No
COG1176	ABC-type spermidine/putrescine transport, permease component I	78	72	33	-3.32226	0.00089	Yes	-2.42376	0.01536	No
COG0601	ABC-type dipeptide/oligopeptide/nickel transport, permease components	251	176	63	-2.72669	0.0064	Yes	0.13412	0.89331	No
COG4608	ABC-type oligopeptide transport, ATPase component	131	99	28	-2.57501	0.01002	Yes	0.86268	0.38831	No
COG1173	ABC-type dipeptide/oligopeptide/nickel transport, permease components	212	146	86	-2.32485	0.02008	No	-3.60919	0.00031	Yes
COG1115	Na+/alanine symporter	129	28	17	4.34046	1.4E-05	Yes	2.57083	0.01015	No
COG0834	ABC-type amino acid transport/signal transduction, periplasmic component	507	145	85	6.68402	2.3E-11	Yes	3.6078	0.00031	Yes
**Amino acid and amine oxidation**										
COG1012	NAD-dependent aldehyde dehydrogenases	95	132	55	-7.07674	1.5E-12	Yes	-4.8225	1.4E-06	Yes
COG3546	Mn-containing catalase	31	0	0	2.76694	0.00566	Yes	2.03804	0.04155	No
COG0753	Catalase	39	0	0	3.00257	0.00268	Yes	2.27132	0.02313	No
COG0665	Glycine/D-amino acid oxidases (deaminating)	76	3	4	4.43312	9.3E-06	Yes	3.08233	0.00205	Yes
pfam07833	Cu_amine_oxidN1, copper amine oxidase	167	6	5	6.49891	8.1E-11	Yes	5.28619	1.2E-07	Yes
COG2414	Aldehyde:ferredoxin oxidoreductase	198	17	15	7.25153	4.1E-13	Yes	4.54519	5.5E-06	Yes
**Ammonia transport and assimilation**										
COG0004	Ammonia permease	129	117	27	-4.11561	3.9E-05	Yes	0.94786	0.3432	No
COG0069	Glutamate synthase domain 2	172	127	57	-2.73268	0.00628	Yes	-1.69523	0.09003	No
COG0174	Glutamine synthetase	241	10	4	7.93291	2.1E-15	Yes	5.42699	5.7E-08	Yes

Function ID	Function description	Estimated gene copies[a]			*A. wheeleri* vs. laboratory *N. corniger*			*Amitermes* vs. Costa Rican *Nasutitermes* sp.		
		Ami[b]	Lab Nas[b]	CR Nas[b]	Z-LOR[c]	p-value	Signifi-cant[d]	Z-LOR[c]	p-value	Signifi-cant[d]
**Urease**										
COG0804	Urea amidohydrolase (urease) alpha subunit	12	40	26	-5.55089	2.8E-08	Yes	-6.12284	9.2E-10	Yes
COG2371	Urease accessory protein UreE	0	9	11	-2.67544	0.00746	Yes	-3.60133	0.00032	Yes
COG0830	Urease accessory protein UreF	3	9	16	-2.5823	0.00981	Yes	-4.82805	1.4E-06	Yes
COG0831	Urea amidohydrolase (urease) gamma subunit	1	8	8	-2.54782	0.01084	Yes	-3.24606	0.00117	Yes
	**Sum of all COGs involved**	**45**	**83**	**93**	**-6.70647**	**2E-11**	**Yes**	**-11.6002**	**4.1E-31**	**Yes**
Amino acid biosynthesis										
**Arginine biosynthesis**										
COG0002	Acetylglutamate semialdehyde dehydrogenase	87	99	26	-5.11931	3.1E-07	Yes	-0.69639	0.48619	No
COG1364	N-acetylglutamate synthase (N-acetylornithine aminotransferase)	138	111	30	-3.17893	0.00148	Yes	0.80754	0.41936	No
COG0137	Argininosuccinate synthase	143	111	42	-2.92257	0.00347	Yes	-0.78799	0.4307	No
**Histidine biosynthesis**										
COG0040	ATP phosphoribosyltransferase	111	90	34	-2.9136	0.00357	Yes	-0.91986	0.35765	No
COG0131	Imidazoleglycerol-phosphate dehydratase	73	62	38	-2.66143	0.00778	Yes	-3.55282	0.00038	Yes
COG0139	Phosphoribosyl-AMP cyclohydrolase	30	31	24	-2.56037	0.01046	Yes	-4.16415	3.1E-05	Yes
COG1387	Histidinol phosphatase and related hydrolases of the PHP family	139	43	57	3.1549	0.00161	Yes	-3.00199	0.00268	Yes
**Isoleucine biosynthesis**										
COG0059	Ketol-acid reductoisomerase	85	105	32	-5.71894	1.1E-08	Yes	-1.86399	0.06232	No
COG0129	Dihydroxyacid dehydratase/phosphogluconate dehydratase	128	133	85	-5.34206	9.2E-08	Yes	-6.82119	9E-12	Yes
COG0028	Thiamine pyrophosphate-requiring enzymes	314	220	113	-3.04042	0.00236	Yes	-3.11458	0.00184	Yes
COG1171	Threonine dehydratase	65	15	14	2.94487	0.00323	Yes	0.5835	0.55956	No
	**Sum of all COGs involved**	**805**	**604**	**288**	**-6.36242**	**2E-10**	**Yes**	**-5.1355**	**2.8E-07**	**Yes**
**Leucine biosynthesis**										
COG0059	Ketol-acid reductoisomerase	85	105	32	-5.71894	1.1E-08	Yes	-1.86399	0.06232	No
COG0473	Isocitrate/isopropylmalate dehydrogenase	111	121	67	-5.39748	6.8E-08	Yes	-5.55151	2.8E-08	Yes
COG0129	Dihydroxyacid dehydratase/phosphogluconate dehydratase	128	133	85	-5.34206	9.2E-08	Yes	-6.82119	9E-12	Yes
COG0066	3-isopropylmalate dehydratase small subunit	30	52	36	-5.11662	3.1E-07	Yes	-6.25371	4E-10	Yes
COG0028	Thiamine pyrophosphate-requiring enzymes	314	220	113	-3.04042	0.00236	Yes	-3.11458	0.00184	Yes
COG0065	3-isopropylmalate dehydratase large subunit	144	107	45	-2.55431	0.01064	Yes	-1.17309	0.24076	No
	**Sum of all COGs involved**	**1440**	**1120**	**535**	**-9.5164**	**1.8E-21**	**Yes**	**-7.72287**	**1.1E-14**	**Yes**

Function ID	Function description	Estimated gene copies[a]			*A. wheeleri* vs. laboratory *N. corniger*			*Amitermes* vs. Costa Rican *Nasutitermes* sp.		
		Ami[b]	Lab Nas[b]	CR Nas[b]	Z-LOR[c]	p-value	Signifi-cant[d]	Z-LOR[c]	p-value	Signifi-cant[d]
**Methionine biosynthesis**										
COG1410	Methionine synthase I, cobalamin-binding domain	177	197	71	-7.05189	1.8E-12	Yes	-3.2044	0.00135	Yes
COG0626	Cystathionine beta-lyases/cystathionine gamma-synthases	94	118	32	-6.15222	7.6E-10	Yes	-1.39706	0.16239	No
COG0620	Methionine synthase II (cobalamin-independent)	0	5	9	-2.03784	0.04157	No	-3.37804	0.00073	Yes
	**Sum of all COGs involved**	**1420**	**969**	**340**	**-5.94634**	**2.7E-09**	**Yes**	**0.82445**	**0.40968**	**No**
**Phenylalanine/tyrosine biosynthesis**										
COG0169	Shikimate 5-dehydrogenase	86	84	51	-3.90799	9.3E-05	Yes	-4.75912	1.9E-06	Yes
COG0077	Prephenate dehydratase	76	67	53	-2.96541	0.00302	Yes	-5.6059	2.1E-08	Yes
COG0710	3-dehydroquinate dehydratase	41	42	22	-2.94728	0.00321	Yes	-2.80375	0.00505	Yes
	**Sum of all COGs involved**	**1029**	**706**	**456**	**-5.17658**	**2.3E-07**	**Yes**	**-10.0865**	**6.3E-24**	**Yes**
**Tryptophan biosynthesis**										
COG0512	Anthranilate/para-aminobenzoate synthases component II	55	68	32	-4.60552	4.1E-06	Yes	-3.69697	0.00022	Yes
COG0547	Anthranilate phosphoribosyltransferase	120	117	27	-4.6009	4.2E-06	Yes	0.60214	0.54708	No
COG0159	Tryptophan synthase alpha chain	75	83	55	-4.54696	5.4E-06	Yes	-5.93449	2.9E-09	Yes
COG0147	Anthranilate/para-aminobenzoate synthases component I	93	92	41	-4.16359	3.1E-05	Yes	-2.90469	0.00368	Yes
COG0169	Shikimate 5-dehydrogenase	86	84	51	-3.90799	9.3E-05	Yes	-4.75912	1.9E-06	Yes
COG0135	Phosphoribosylanthranilate isomerase	43	46	39	-3.25483	0.00113	Yes	-5.72517	1E-08	Yes
COG0134	Indole-3-glycerol phosphate synthase	85	75	29	-3.14243	0.00168	Yes	-1.33981	0.18031	No
COG0710	3-dehydroquinate dehydratase	41	42	22	-2.94728	0.00321	Yes	-2.80375	0.00505	Yes
COG0133	Tryptophan synthase beta chain	134	100	53	-2.49968	0.01243	No	-2.68705	0.00721	Yes
	**Sum of all COGs involved**	**1440**	**1145**	**583**	**-10.1353**	**3.9E-24**	**Yes**	**-9.72277**	**2.4E-22**	**Yes**
**Valine biosynthesis**										
COG0059	Ketol-acid reductoisomerase	85	105	32	-5.71894	1.1E-08	Yes	-1.86399	0.06232	No
COG0129	Dihydroxyacid dehydratase/phosphogluconate dehydratase	128	133	85	-5.34206	9.2E-08	Yes	-6.82119	9E-12	Yes
COG0436	Aspartate/tyrosine/aromatic aminotransferase	520	350	153	-3.28706	0.00101	Yes	-1.52434	0.12742	No
COG0028	Thiamine pyrophosphate-requiring enzymes	314	220	113	-3.04042	0.00236	Yes	-3.11458	0.00184	Yes
	**Sum of all COGs involved**	**1260**	**939**	**427**	**-7.79551**	**6.4E-15**	**Yes**	**-5.33275**	**9.7E-08**	**Yes**
Vitamin biosynthesis										
**Thiamine biosynthesis**										
COG1060	Thiamine biosynthesis enzyme ThiH and related uncharacterized enzymes	110	162	50	-8.17842	2.9E-16	Yes	-3.372	0.00075	Yes
COG0422	Thiamine biosynthesis protein ThiC	40	47	9	-3.64565	0.00027	Yes	0.34759	0.72815	No
COG0352	Thiamine monophosphate synthase	37	43	33	-3.44837	0.00056	Yes	-5.21717	1.8E-07	Yes

Function ID	Function description	Estimated gene copies[a]			*A. wheeleri* vs. laboratory *N. corniger*			*Amitermes* vs. Costa Rican *Nasutitermes* sp.		
		Ami[b]	Lab Nas[b]	CR Nas[b]	Z-LOR[c]	p-value	Signifi-cant[d]	Z-LOR[c]	p-value	Signifi-cant[d]
COG0476	Dinucleotide-utilizing enzymes in molybdopterin/thiamine biosynthesis	97	72	49	-2.08872	0.03673	No	-3.88391	0.0001	Yes
COG0351	Hydroxymethylpyrimidine/phosphomethylpyrimidine kinase	47	39	29	-2.01451	0.04396	No	-3.72964	0.00019	Yes
	**Sum of all COGs involved**	**523**	**488**	**240**	**-8.90767**	**5.2E-19**	**Yes**	**-7.71726**	**1.2E-14**	**Yes**
**Biotin**										
COG0502	Biotin synthase and related enzymes	53	63	44	-4.2699	2E-05	Yes	-5.77369	7.8E-09	Yes
COG0161	Adenosylmethionine-8-amino-7-oxononanoate aminotransferase	22	28	5	-3.03302	0.00242	Yes	0.23854	0.81146	No
COG0156	7-keto-8-aminopelargonate synthetase and related enzymes	96	27	16	2.96429	0.00303	Yes	1.58653	0.11262	No
	**Sum of all COGs involved**	**280**	**186**	**100**	**-2.32779**	**0.01992**	**Yes**	**-3.0082**	**0.00263**	**Yes**
**Cobalamin biosynthesis**										
COG0310	ABC-type Co2+ transport system, permease component	7	40	6	-5.77504	7.7E-09	Yes	-2.17365	0.02973	No
COG1429	Cobalamin biosynthesis protein CobN and related Mg-chelatases	13	25	10	-3.73424	0.00019	Yes	-2.61781	0.00885	No
**Inorganic phosphate metabolism**										
COG1785	Alkaline phosphatase	40	54	18	-4.42507	9.6E-06	Yes	-1.9906	0.04653	No
COG1283	Na+/phosphate symporter	163	122	43	-2.78402	0.00537	Yes	-0.18018	0.85701	No
COG0855	Polyphosphate kinase	191	139	76	-2.73772	0.00619	Yes	-3.25971	0.00112	Yes
**Sugar transporter**										
COG4213	ABC-type xylose transport, periplasmic component	91	113	26	-5.96143	2.5E-09	Yes	-0.49776	0.61866	No
COG0395	ABC-type sugar transport, permease component	783	357	190	2.5488	0.01081	Yes	0.65247	0.5141	No
COG3090	TRAP-type C4-dicarboxylate transport, small permease component	35	6	9	2.58176	0.00983	Yes	-0.0142	0.98867	No
COG1653	ABC-type sugar transport, periplasmic component	729	327	197	2.69079	0.00713	Yes	-0.68551	0.49302	No
COG4211	ABC-type glucose/galactose transport, permease component	46	9	0	2.76721	0.00565	Yes	2.439	0.01473	No
COG2211	Na+/melibiose symporter and related transporters	204	68	47	3.39866	0.00068	Yes	0.64641	0.51802	No
COG1129	ABC-type sugar transport, ATPase component	578	232	64	3.73491	0.00019	Yes	6.36224	2E-10	Yes
COG1175	ABC-type sugar transport, permease components	525	205	101	3.85909	0.00011	Yes	2.62438	0.00868	No
COG0738	Fucose permease	104	8	13	5.29397	1.2E-07	Yes	2.43445	0.01491	No
COG1172	Ribose/xylose/arabinose/galactoside ABC transport, permease components	249	57	34	5.80172	6.6E-09	Yes	3.43462	0.00059	Yes
	**Sum of all COGs involved**	**4651**	**2034**	**919**	**7.51814**	**5.6E-14**	**Yes**	**6.76013**	**1.4E-11**	**Yes**
**Glycolysis**										
COG0205	6-phosphofructokinase	323	271	100	-5.43506	5.5E-08	Yes	-1.66977	0.09497	No
COG0837	Glucokinase	6	15	6	-3.18651	0.00144	Yes	-2.36158	0.0182	No
COG0696	Phosphoglyceromutase	216	154	36	-2.6995	0.00694	Yes	2.38035	0.0173	No

Function ID	Function description	Estimated gene copies[a]			*A. wheeleri* vs. laboratory *N. corniger*			*Amitermes* vs. Costa Rican *Nasutitermes* sp.		
		Ami[b]	Lab Nas[b]	CR Nas[b]	Z-LOR[c]	p-value	Signifi-cant[d]	Z-LOR[c]	p-value	Signifi-cant[d]
COG3635	Predicted phosphoglycerate mutase, AP superfamily	97	7	10	5.12615	3E-07	Yes	2.73646	0.00621	Yes
**Pentose phosphate pathway**										
COG0021	Transketolase	104	163	61	-8.54722	1.3E-17	Yes	-5.14751	2.6E-07	Yes
COG0120	Ribose 5-phosphate isomerase	27	46	32	-4.76749	1.9E-06	Yes	-5.86838	4.4E-09	Yes
COG0362	6-phosphogluconate dehydrogenase	85	81	27	-3.70193	0.00021	Yes	-0.98074	0.32672	No
COG0363	6-phosphogluconolactonase/Glucosamine-6-P isomerase/deaminase	82	23	13	2.74817	0.00599	Yes	1.60259	0.10902	No
COG0698	Ribose 5-phosphate isomerase RpiB	79	13	9	3.94842	7.9E-05	Yes	2.2993	0.02149	No
	**Sum of all COGs involved**	**648**	**449**	**207**	**-4.27707**	**1.9E-05**	**Yes**	**-2.99373**	**0.00276**	**Yes**
**Hydrogenase**										
COG4624	Iron only hydrogenase large subunit, C-terminal domain	341	225	163	-2.41317	0.01581	No	-6.57231	5E-11	Yes
pfam02906	Fe_hyd_lg_C	364	245	217	-2.79211	0.00524	Yes	-6.91755	4.6E-12	Yes
COG3261	Ni,Fe-hydrogenase III large subunit	39	5	1	3.01321	0.00258	Yes	2.27132	0.02313	No
**Pyruvate decarboxylation**										
COG0280	Phosphotransacetylase	103	93	26	-3.64155	0.00027	Yes	0.06017	0.95202	No
**Homoacetogenesis**										
COG1456	CO dehydrogenase/acetyl-CoA synthase gamma subunit (corrinoid Fe-S protein)	49	70	39	-5.25996	1.4E-07	Yes	-5.29079	1.2E-07	Yes
COG1152	CO dehydrogenase/acetyl-CoA synthase alpha subunit	12	26	8	-4.00056	6.3E-05	Yes	-2.09887	0.03583	No
COG1151	6Fe-6S prismane cluster-containing protein	46	53	42	-3.79454	0.00015	Yes	-5.96318	2.5E-09	Yes
COG2069	CO dehydrogenase/acetyl-CoA synthase delta subunit (corrinoid Fe-S protein)	19	25	11	-2.94847	0.00319	Yes	-2.15624	0.03107	No
COG1614	CO dehydrogenase/acetyl-CoA synthase beta subunit	33	33	34	-2.53041	0.01139	No	-5.70236	1.2E-08	Yes
COG0243	Anaerobic dehydrogenases, typically selenocysteine-containing	75	7	5	4.42522	9.6E-06	Yes	2.91156	0.0036	Yes
COG1882	Pyruvate-formate lyase	207	33	30	6.47531	9.5E-11	Yes	2.90913	0.00362	Yes
Cell motility										
**Chemotaxis**										
COG0840	Methyl-accepting chemotaxis protein	825	1878	565	-34.7592	1E-264	Yes	-18.0801	4.6E-73	Yes
COG1352	Methylase of chemotaxis methyl-accepting proteins	183	143	40	-3.3725	0.00074	Yes	0.90086	0.36766	No
COG1776	Chemotaxis protein CheC, inhibitor of MCP methylation	110	29	16	3.40341	0.00067	Yes	2.11008	0.03485	No
	**Sum of all COGs involved**	**2055**	**2578**	**847**	**-29.1401**	**1E-186**	**Yes**	**-12.1467**	**6E-34**	**Yes**
**Flagellum structure and biogenesis**										
COG1344	Flagellin and related hook-associated proteins	617	533	225	-8.07159	6.9E-16	Yes	-4.5605	5.1E-06	Yes

Function ID	Function description	Estimated gene copies[a]			*A. wheeleri* vs. laboratory *N. corniger*			*Amitermes* vs. Costa Rican *Nasutitermes* sp.		
		Ami[b]	Lab Nas[b]	CR Nas[b]	Z-LOR[c]	p-value	Signifi-cant[d]	Z-LOR[c]	p-value	Signifi-cant[d]
COG1987	Flagellar biosynthesis pathway, component FliQ	41	52	17	-4.12089	3.8E-05	Yes	-1.67477	0.09398	No
COG1580	Flagellar basal body-associated protein	28	41	23	-4.0967	4.2E-05	Yes	-4.14634	3.4E-05	Yes
COG3144	Flagellar hook-length control protein	31	41	14	-3.79215	0.00015	Yes	-1.76564	0.07746	No
COG1419	Flagellar GTP-binding protein	87	81	33	-3.57222	0.00035	Yes	-1.92771	0.05389	No
COG1749	Flagellar hook protein FlgE	167	135	46	-3.5454	0.00039	Yes	-0.44503	0.6563	No
COG1536	Flagellar motor switch protein	218	168	125	-3.53148	0.00041	Yes	-7.19951	6E-13	Yes
COG1157	Flagellar biosynthesis/type III secretory pathway ATPase	162	130	62	-3.42239	0.00062	Yes	-2.69938	0.00695	Yes
COG1886	Flagellar motor switch/type III secretory pathway protein	53	54	39	-3.31863	0.0009	Yes	-5.00956	5.5E-07	Yes
COG1766	Flagellar biosynthesis/type III secretory pathway lipoprotein	174	131	35	-2.93211	0.00337	Yes	1.29749	0.19446	No
COG1317	Flagellar biosynthesis/type III secretory pathway protein	73	57	40	-2.12466	0.03362	No	-3.87353	0.00011	Yes
COG1868	Flagellar motor switch protein	151	106	71	-2.124	0.03367	No	-4.23255	2.3E-05	Yes
COG1815	Flagellar basal body protein	74	57	34	-2.05377	0.04	No	-2.8276	0.00469	Yes
COG1334	Uncharacterized flagellar protein FlaG	25	24	24	-2.03694	0.04166	No	-4.62859	3.7E-06	Yes
COG1843	Flagellar hook capping protein	163	57	58	2.78105	0.00542	Yes	-2.15998	0.03077	No
	**Sum of all COGs involved**	**4011**	**2803**	**1401**	**-11.1036**	**1.2E-28**	**Yes**	**-10.6674**	**1.4E-26**	**Yes**
**Pilus**										
COG3063	Tfp pilus assembly protein PilF	217	207	148	-5.92919	3E-09	Yes	-9.20561	3.4E-20	Yes
COG4972	Tfp pilus assembly protein, ATPase PilM	31	38	6	-3.41528	0.00064	Yes	0.62513	0.53188	No
COG3166	Tfp pilus assembly protein PilN	2	11	1	-3.02806	0.00246	Yes	-0.54729	0.58418	No
COG3188	P pilus assembly protein, porin PapC	11	18	13	-2.91453	0.00356	Yes	-3.73605	0.00019	Yes
COG2805	Tfp pilus assembly protein, pilus retraction ATPase PilT	139	45	24	2.94472	0.00323	Yes	1.77837	0.07534	No
COG2804	Type II secretory pathway, ATPase PulE/Tfp pilus assembly pathway	226	80	45	3.19613	0.00139	Yes	1.53491	0.1248	No
	**Sum of all COGs involved**	**719**	**445**	**245**	**-2.4794**	**0.01316**	**Yes**	**-4.10584**	**4E-05**	**Yes**
**Defense**										
COG4096	Type I site-specific restriction-modification system, R (restriction) subunit	86	119	25	-6.69779	2.1E-11	Yes	-0.56326	0.57326	No
COG4452	Inner membrane protein involved in colicin E2 resistance	47	76	18	-5.94765	2.7E-09	Yes	-1.45642	0.14528	No
COG0841	Cation/multidrug efflux pump	840	580	206	-4.68651	2.8E-06	Yes	0.5424	0.58754	No
COG0286	Type I restriction-modification system methyltransferase subunit	477	216	81	2.06695	0.03874	No	3.41188	0.00065	Yes
COG4823	Abortive infection bacteriophage resistance protein	88	24	6	2.93771	0.00331	Yes	3.13399	0.00172	Yes
COG2720	Uncharacterized vancomycin resistance protein	45	2	0	3.44673	0.00057	Yes	2.41668	0.01566	No
COG1619	Uncharacterized proteins, homologs of microcin C7 resistance protein MccF	62	1	0	3.47656	0.00051	Yes	2.7419	0.00611	Yes
COG0732	Restriction endonuclease S subunits	178	50	42	4.04285	5.3E-05	Yes	0.47052	0.63799	No
COG1403	Restriction endonuclease	75	9	10	4.24516	2.2E-05	Yes	1.93544	0.05294	No
COG1136	ABC-type antimicrobial peptide transport system, ATPase component	662	223	108	6.01582	1.8E-09	Yes	4.33898	1.4E-05	Yes

Function ID	Function description	Estimated gene copies[a]			*A. wheeleri* vs. laboratory *N. corniger*			*Amitermes* vs. Costa Rican *Nasutitermes* sp.		
		Ami[b]	Lab Nas[b]	CR Nas[b]	Z-LOR[c]	p-value	Signifi-cant[d]	Z-LOR[c]	p-value	Signifi-cant[d]
COG1131	ABC-type multidrug transport system, ATPase component	740	238	107	6.87338	6.3E-12	Yes	5.52279	3.3E-08	Yes
COG2274	ABC-type bacteriocin/lantibiotic exporters	590	147	57	8.32696	8.3E-17	Yes	7.02703	2.1E-12	Yes
	**Sum of all COGs involved**	**6768**	**3186**	**1247**	**5.86928**	**4.4E-09**	**Yes**	**10.2527**	**1.2E-24**	**Yes**
**Sporulation**										
pfam05036	SPOR : Sporulation related domain	124	95	61	-2.64127	0.00826	Yes	-2.56194	0.01041	No
pfam09546	Spore_III_AE : Stage III sporulation protein AE (spore_III_AE)	27	2	1	2.69688	0.007	Yes	2.14659	0.03183	No
pfam06686	SpoIIIAC : Stage III sporulation protein AC/AD protein family	30	1	2	2.72976	0.00634	Yes	2.18841	0.02864	No
pfam08769	Spo0A_C : Sporulation initiation factor Spo0A C terminal	35	0	0	2.88809	0.00388	Yes	2.41132	0.01589	No
pfam03862	SpoVA : SpoVA protein	34	3	2	2.99099	0.00278	Yes	2.36854	0.01786	No
pfam07451	SpoVAD : Stage V sporulation protein AD (SpoVAD)	44	1	0	3.12265	0.00179	Yes	2.64452	0.00818	Yes
pfam07454	SpoIIP : Stage II sporulation protein P (SpoIIP)	45	0	0	3.14565	0.00166	Yes	2.66741	0.00764	Yes
pfam02650	HTH_WhiA : Sporulation Regulator WhiA C terminal domain	52	0	0	3.29351	0.00099	Yes	2.81456	0.00488	Yes
pfam09547	Spore_IV_A : Stage IV sporulation protein A (spore_IV_A)	57	0	0	3.38727	0.00071	Yes	2.90792	0.00364	Yes
pfam05580	SpoIVB peptidase S55: SpoIVB peptidase S55	51	2	0	3.62416	0.00029	Yes	2.7948	0.00519	Yes
pfam01943	Polysacc_synt: Spore formation	121	20	6	4.86321	1.2E-06	Yes	4.52949	5.9E-06	Yes
	**Sum of all pfams involved**	**1395**	**458**	**262**	**6.26254**	**3.8E-10**	**Yes**	**6.26254**	**3.8E-10**	**Yes**
**Germination**										
pfam03418	Peptidase_A25 : Germination protease	30	0	0	2.72976	0.00634	Yes	2.25409	0.02419	No
pfam03323	GerA : Bacillus/Clostridium GerA spore germination protein	78	1	1	3.70692	0.00021	Yes	3.22645	0.00125	Yes
	**Sum of all pfams involved**	**202**	**31**	**20**	**5.52594**	**3.3E-08**	**Yes**	**5.52594**	**3.3E-08**	**Yes**

[a]Estimated gene copy is the number of genes weighted by their individual read depth, which reflects population abundance.
[b]Ami = *A. wheeleri*; Lab Nas = Laboratory *N. corniger*; CR Nas = Costa Rican *Nasutitermes* sp.
[c]Z-LOR is the calculated as the natural logarithm of odds ratio (LOR) divided by the standard error of LOR. P-value is calculated from Z-LOR.
[d]Statistical significance was determined after adjusting for multiple hypothesis test, with a false discovery rate cutoff of 0.05.
[e]Functions involved in this pathway were added up, although only the individual function with a statistical differences was shown.
[f]Color indication: [dark green] = function statistically overrepresented in *A. wheeleri* in both comparisons; [light green] = function statistically overrepresented in *A. wheeleri* in only one comparison, and the overrepresentation is supported by the other comparison (Z-LOR > 2); [pale green] = function statistically overrepresented in *A. wheeleri* in only one comparison, and the overrepresentation is neither supported nor against by the other comparison (abs(Z-LOR) < 2); [dark orange] = function statistically underrepresented in *A. wheeleri* in both comparisons; [light orange] = function statistically underrepresented in *A. wheeleri* in only one comparison, and the underrepresentation is supported by the other comparison (Z-LOR < -2); [pale orange] = function statistically underrepresented in *A. wheeleri* in only one comparison, and the underrepresentation is neither supported nor against by the other comparison; [grey] = function with statistical differences but conflicting between the two comparisons.
