# Supplementary material for: Comparative Metagenomic and Metatranscriptomic Analysis of Hindgut Paunch Microbiota in Wood- and Dung-Feeding Higher Termites
Source: PLoS One. 2013 Apr 12;8(4):e61126. doi: 10.1371/journal.pone.0061126 (PMC3625147; doi:10.1371/journal.pone.0061126)
Supplement: Table S5 — Major functions differentially expressed between A. wheeleri and N. corniger metatranscriptomes (PDF) [file pone.0061126.s013.pdf]

Table S5. Major functions differentially expressed between *A. wheeleri* and *N. corniger* metatranscriptomes[a]

Function ID	Function description	Expression level		*A. wheeleri* vs. Lab *N. corniger*		
		Ami	Lab Nas	Z-LOR	p-value	Significant
**Glycoside hydrolases in plant cell wall degradation**						
pfam01055	Glyco_hydro_31: Oligosaccharide degrading	17	0	2.53	1.14E-02	Yes
pfam00933	Glyco_hydro_3: Oligosaccharide degrading	82	0	4.15	3.30E-05	Yes
pfam01270	Glyco_hydro_8: Hemicellulase	0	36	-3.76	1.67E-04	Yes
pfam07745	Glyco_hydro_53: Hemicellulase	18	2	2.64	8.34E-03	Yes
pfam00331	Glyco_hydro_10: Hemicellulase	90	8	5.94	2.91E-09	Yes
pfam00457	Glyco_hydro_11: Hemicellulase	1013	70	19.87	7.72E-88	Yes
pfam00150	Cellulase, GH5: Cellulase	132	519	-16.48	4.85E-61	Yes
pfam02015	Glyco_hydro_45: Cellulase	27	4	3.13	1.73E-03	Yes
COG3534	Alpha-L-arabinofuranosidase: Debranching	58	70	-2.84	4.54E-03	Yes
**Cohesin and dockerin**						
pfam09603	Fib_succ_major, corresponding to TIGR02145	100	574	-18.34	4.13E-75	Yes
**Nitrogen fixation**						
COG2710	Nitrogenase molybdenum-iron protein, alpha and beta chains	13	183	-10.33	5.42E-25	Yes
COG0347	Nitrogen regulatory protein PII	2	139	-6.40	1.52E-10	Yes
COG1348	Nitrogenase subunit NifH (ATPase)	2	87	-5.72	1.07E-08	Yes
COG5000	Signal transduction histidine kinase in N2 fixation/metabolism regulation	0	20	-3.23	1.23E-03	Yes
	**Sum of all COGs involved**	**17**	**429**	**-14.46**	**2.24E-47**	**Yes**
**Amino acid transport**						
COG0601	ABC-type dipeptide/oligopeptide/nickel transport, permease components	47	133	-8.00	1.26E-15	Yes
COG0834	ABC-type amino acid transport/signal transduction, periplasmic component	27	51	-4.00	6.34E-05	Yes
COG1176	ABC-type spermidine/putrescine transport, permease component I	0	25	-3.47	5.28E-04	Yes
COG0591	Na+/proline symporter	3	11	-2.48	1.32E-02	Yes
COG4177	ABC-type branched-chain amino acid transport, permease component	20	1	2.62	8.90E-03	Yes
COG0559	Branched-chain amino acid ABC-type transport, permease components	26	4	2.90	3.75E-03	Yes
COG0411	ABC-type branched-chain amino acid transport, ATPase component	35	0	3.20	1.40E-03	Yes
COG0444	ABC-type dipeptide/oligopeptide/nickel transport, ATPase component	99	37	3.47	5.15E-04	Yes
COG3842	ABC-type spermidine/putrescine transport, ATPase components	70	17	4.07	4.72E-05	Yes
COG1115	Na+/alanine symporter	68	3	4.76	1.97E-06	Yes
COG4608	ABC-type oligopeptide transport, ATPase component	87	3	5.20	2.00E-07	Yes
COG0683	ABC-type branched-chain amino acid transport, periplasmic component	199	46	7.03	2.01E-12	Yes
COG4166	ABC-type oligopeptide transport, periplasmic component	354	116	7.50	6.44E-14	Yes

Function ID	Function description	Expression level		*A. wheeleri* vs. Lab *N. corniger*		
		Ami	Lab Nas	Z-LOR	p-value	Significant
COG0747	ABC-type dipeptide transport, periplasmic component	884	247	13.42	4.82E-41	Yes
	**Sum of all COGs involved**	**2020**	**758**	**15.33**	**4.51E-53**	**Yes**
**Amino acid and amine oxidation**						
COG0753	Catalase	34	0	3.17	1.55E-03	Yes
COG3546	Mn-containing catalase	59	0	3.73	1.90E-04	Yes
COG2414	Aldehyde:ferredoxin oxidoreductase	148	27	6.63	3.38E-11	Yes
pfam07833	Cu_amine_oxidN1, copper amine oxidase	724	17	14.40	5.38E-47	Yes
**Ammonia transport and assimilation**						
COG0004	Ammonia permease	13	133	-9.10	9.32E-20	Yes
COG3968	Protein related to glutamine synthetase	20	124	-8.89	6.14E-19	Yes
COG0069	Glutamate synthase domain 2	6	75	-6.70	2.08E-11	Yes
	**Sum of all COGs involved**	**69**	**354**	**-15.03**	**4.62E-51**	**Yes**
Amino acid biosynthesis						
**Arginine biosynthesis**						
COG0458	Carbamoylphosphate synthase large subunit (split gene in MJ)	46	16	2.55	1.07E-02	Yes
COG0548	Acetylglutamate kinase	27	2	3.12	1.80E-03	Yes
	**Sum of all COGs involved**	**192**	**70**	**4.82**	**1.45E-06**	**Yes**
**Isoleucine biosynthesis**						
COG0059	Ketol-acid reductoisomerase	46	122	-7.47	8.11E-14	Yes
COG0440	Acetolactate synthase, small (regulatory) subunit	8	30	-4.12	3.86E-05	Yes
COG0028	Thiamine pyrophosphate-requiring enzymes	105	50	2.48	1.30E-02	Yes
	**Sum of all COGs involved**	**197**	**233**	**-5.23**	**1.68E-07**	**Yes**
**Leucine biosynthesis**						
COG0059	Ketol-acid reductoisomerase	46	122	-7.47	8.11E-14	Yes
COG0440	Acetolactate synthase, small (regulatory) subunit	8	30	-4.12	3.86E-05	Yes
COG0028	Thiamine pyrophosphate-requiring enzymes	105	50	2.48	1.30E-02	Yes
	**Sum of all COGs involved**	**299**	**320**	**-5.05**	**4.31E-07**	**Yes**
**Valine biosynthesis**						
COG0059	Ketol-acid reductoisomerase	46	122	-7.47	8.11E-14	Yes
COG0440	Acetolactate synthase, small (regulatory) subunit	8	30	-4.12	3.86E-05	Yes
COG0028	Thiamine pyrophosphate-requiring enzymes	105	50	2.48	1.30E-02	Yes
	**Sum of all COGs involved**	**226**	**261**	**-5.31**	**1.09E-07**	**Yes**

Function ID	Function description	Expression level		*A. wheeleri* vs. Lab *N. corniger*		
		Ami	Lab Nas	Z-LOR	p-value	Significant
Vitamin biosynthesis						
**Thiamine biosynthesis**						
COG0422	Thiamine biosynthesis protein ThiC	4	17	-3.17	1.51E-03	Yes
COG0352	Thiamine monophosphate synthase	5	14	-2.58	9.82E-03	Yes
	**Sum of all COGs involved**	**34**	**55**	**-3.75**	**1.75E-04**	**Yes**
**Inorganic phosphate metabolism**						
COG0226	ABC-type phosphate transport system, periplasmic component	211	344	-9.23	2.78E-20	Yes
COG0581	ABC-type phosphate transport system, permease component	7	175	-9.18	4.33E-20	Yes
COG0704	Phosphate uptake regulator	5	219	-9.07	1.24E-19	Yes
COG1785	Alkaline phosphatase	56	155	-8.56	1.09E-17	Yes
COG1117	ABC-type phosphate transport system, ATPase component	21	86	-7.09	1.31E-12	Yes
COG0855	Polyphosphate kinase	0	65	-4.46	8.30E-06	Yes
COG0248	Exopolyphosphatase	3	25	-3.99	6.69E-05	Yes
COG0573	ABC-type phosphate transport system, permease component	50	66	-3.17	1.55E-03	Yes
COG1283	Na+/phosphate symporter	26	5	2.73	6.32E-03	Yes
**Sugar transporter**						
COG1653	ABC-type sugar transport system, periplasmic component	711	733	-6.63	3.47E-11	Yes
COG0395	ABC-type sugar transport system, permease component	59	122	-6.58	4.77E-11	Yes
COG4214	ABC-type xylose transport system, permease component	22	39	-3.33	8.63E-04	Yes
COG4211	ABC-type glucose/galactose transport system, permease component	25	0	2.85	4.41E-03	Yes
COG1593	TRAP-type C4-dicarboxylate transport system, large permease component	27	1	2.93	3.42E-03	Yes
COG3839	ABC-type sugar transport systems, ATPase components	433	246	3.14	1.68E-03	Yes
COG1172	Ribose/xylose/arabinose/galactoside ABC transport, permease components	63	2	4.37	1.27E-05	Yes
COG1638	TRAP-type C4-dicarboxylate transport system, periplasmic component	223	1	5.08	3.69E-07	Yes
COG1129	ABC-type sugar transport system, ATPase component	187	21	8.14	3.98E-16	Yes
COG1879	ABC-type sugar transport system, periplasmic component	671	18	13.87	9.15E-44	Yes
**Glycolysis**						
COG0057	Glyceraldehyde-3-phosphate dehydrogenase	487	482	-4.78	1.79E-06	Yes
COG0149	Triosephosphate isomerase	67	94	-4.09	4.22E-05	Yes
COG0191	Fructose/tagatose bisphosphate aldolase	112	118	-2.79	5.26E-03	Yes
COG0166	Glucose-6-phosphate isomerase	34	4	3.45	5.56E-04	Yes
	**Sum of all COGs involved**	**1234**	**1073**	**-4.79**	**1.65E-06**	**Yes**
**Pentose phosphate pathway**						
COG0176	Transaldolase	18	0	2.51	1.22E-02	Yes

Function ID	Function description	Expression level		*A. wheeleri* vs. Lab *N. corniger*		
		Ami	Lab Nas	Z-LOR	p-value	Significant
COG0235	Ribulose-5-phosphate 4-epimerase and related epimerases and aldolases	40	0	3.33	8.60E-04	Yes
COG0362	6-phosphogluconate dehydrogenase	71	1	3.92	8.82E-05	Yes
	**Sum of all COGs involved**	**206**	**21**	**8.51**	**1.79E-17**	**Yes**
**Pyruvate decarboxylation**						
COG0674	Pyruvate:ferredoxin oxidoreductase, alpha subunit	448	250	3.41	6.61E-04	Yes
COG1013	Pyruvate:ferredoxin oxidoreductase, beta subunit	402	150	7.03	2.11E-12	Yes
	**Sum of all COGs involved**	**1056**	**536**	**6.48**	**8.94E-11**	**Yes**
**Homoacetogenesis**						
COG0685	5,10-methylenetetrahydrofolate reductase	27	78	-6.17	6.91E-10	Yes
COG2069	CO dehydrogenase/acetyl-CoA synthase delta subunit (corrinoid Fe-S protein)	57	69	-2.83	4.62E-03	Yes
COG1882	Pyruvate-formate lyase	26	0	2.89	3.88E-03	Yes
COG0243	Anaerobic dehydrogenases, typically selenocysteine-containing	56	3	4.41	1.05E-05	Yes
COG3383	Uncharacterized anaerobic dehydrogenase	205	57	6.45	1.12E-10	Yes
COG2759	Formyltetrahydrofolate synthetase	657	352	4.70	2.65E-06	Yes
COG0190	5,10-methylene-THF dehydrogenase/Methenyl THF cyclohydrolase	106	24	5.18	2.23E-07	Yes
COG1152	CO dehydrogenase/acetyl-CoA synthase alpha subunit	39	7	3.42	6.34E-04	Yes
COG1456	CO dehydrogenase/acetyl-CoA synthase gamma subunit (corrinoid Fe-S protein)	102	34	3.96	7.58E-05	Yes
COG1614	CO dehydrogenase/acetyl-CoA synthase beta subunit	319	141	4.97	6.85E-07	Yes
	**Sum of all COGs involved**	**1876**	**1014**	**7.25**	**4.24E-13**	**Yes**
Cell motility						
**Chemotaxis**						
COG0840	Methyl-accepting chemotaxis protein	111	1300	-28.33	1.58E-176	Yes
COG1352	Methylase of chemotaxis methyl-accepting proteins	31	131	-8.80	1.31E-18	Yes
COG1871	Chemotaxis protein; stimulates methylation of MCP proteins	30	91	-6.77	1.25E-11	Yes
COG2201	Chemotaxis response regulator	102	173	-6.77	1.29E-11	Yes
COG1776	Chemotaxis protein CheC, inhibitor of MCP methylation	33	0	3.13	1.72E-03	Yes
	**Sum of all COGs involved**	**605**	**1906**	**-32.33**	**2.28E-229**	**Yes**
**Flagellum structure and biogenesis**						
COG1677	Flagellar hook-basal body protein	9	72	-6.78	1.22E-11	Yes
COG1334	Uncharacterized flagellar protein FlaG	103	169	-6.50	8.28E-11	Yes
COG1345	Flagellar capping protein	52	112	-6.46	1.06E-10	Yes
COG1766	Flagellar biosynthesis/type III secretory pathway lipoprotein	13	55	-5.70	1.18E-08	Yes
COG1360	Flagellar motor protein	18	60	-5.66	1.54E-08	Yes
COG1886	Flagellar motor switch/type III secretory pathway protein	23	62	-5.36	8.47E-08	Yes

Function ID	Function description	Expression level		*A. wheeleri* vs. Lab *N. corniger*		
		Ami	Lab Nas	Z-LOR	p-value	Significant
COG1843	Flagellar hook capping protein	63	82	-3.46	5.41E-04	Yes
COG1317	Flagellar biosynthesis/type III secretory pathway protein	4	19	-3.41	6.58E-04	Yes
COG1815	Flagellar basal body protein	13	25	-2.84	4.57E-03	Yes
COG2747	Negative regulator of flagellin synthesis (anti-sigma28 factor)	18	0	2.51	1.22E-02	Yes
COG1157	Flagellar biosynthesis/type III secretory pathway ATPase	56	14	3.58	3.37E-04	Yes
COG1558	Flagellar basal body rod protein	42	4	3.89	9.96E-05	Yes
COG4786	Flagellar basal body rod protein	148	38	5.75	9.07E-09	Yes
COG1344	Flagellin and related hook-associated proteins	9847	5433	18.07	5.94E-73	Yes
	**Sum of all COGs involved**	**10772**	**6402**	**12.88**	**5.57E-38**	**Yes**
**Pilus**						
COG3063	Tfp pilus assembly protein PilF	2	71	-5.42	5.93E-08	Yes
COG4966	Tfp pilus assembly protein PilW	0	58	-4.34	1.42E-05	Yes
COG2804	Type II secretory pathway, ATPase PulE/Tfp pilus assembly pathway, ATPase PilB	64	87	-3.78	1.55E-04	Yes
COG2805	Tfp pilus assembly protein, pilus retraction ATPase PilT	96	39	3.08	2.04E-03	Yes
COG4970	Tfp pilus assembly protein FimT	68	0	3.88	1.06E-04	Yes
COG4968	Tfp pilus assembly protein PilE	93	19	5.06	4.24E-07	Yes
**Defense**						
COG1131	ABC-type multidrug transport system, ATPase component	48	77	-4.29	1.80E-05	Yes
COG0841	Cation/multidrug efflux pump	90	110	-3.64	2.77E-04	Yes
COG1968	Uncharacterized bacitracin resistance protein	3	18	-3.38	7.26E-04	Yes
COG0534	Na+-driven multidrug efflux pump	1	10	-2.50	1.25E-02	Yes
COG1132	ABC-type multidrug transport system, ATPase and permease components	18	2	2.52	1.16E-02	Yes
COG2274	ABC-type bacteriocin/lantibiotic exporters, contain an N-terminal double-glycine peptidas	21	0	2.67	7.66E-03	Yes
COG1136	ABC-type antimicrobial peptide transport system, ATPase component	81	34	2.71	6.82E-03	Yes
**Sporulation**						
pfam04026	SpoVG : SpoVG	61	116	-5.53	3.17E-08	Yes
pfam07228	SpoIIE : Stage II sporulation protein E (SpoIIE)	1	14	-2.77	5.55E-03	Yes
pfam07451	SpoVAD : Stage V sporulation protein AD (SpoVAD)	19	0	2.64	8.18E-03	Yes
pfam04232	SpoVS : Stage V sporulation protein S (SpoVS)	47	0	3.58	3.41E-04	Yes
pfam12116	SpoIIID : Stage III sporulation protein D	72	0	4.02	5.85E-05	Yes
pfam09547	Spore_IV_A : Stage IV sporulation protein A (spore_IV_A)	149	0	4.76	1.94E-06	Yes
	**Sum of all pfams involved**	**421**	**151**	**8.20**	**2.33E-16**	**Yes**

[a]See Table S4 footnotes for explanation. Colors in this table indicate: [green] = function with a statistically higher expression in *A. wheeleri*; [orange] = function with a statistically higher expression in laboratory *N. corniger*.
