# Supplementary material for: Comparative Metagenomic and Metatranscriptomic Analysis of Hindgut Paunch Microbiota in Wood- and Dung-Feeding Higher Termites
Source: PLoS One. 2013 Apr 12;8(4):e61126. doi: 10.1371/journal.pone.0061126 (PMC3625147; doi:10.1371/journal.pone.0061126)
Supplement: Table S6 — Glycoside hydrolase (GH) abundance in metagenomes (PDF) [file pone.0061126.s014.pdf]

Table S6. Glycoside hydrolase (GH) abundance in metagenomes

GH ID	Pfam HMM Name	Pfam Accession	Gene Count[a]		Estimated gene copy[b]	
			A. wheeleri (cow dung)	*N. corniger* (lab colony)	*A. wheeleri* (cow dung)	*N. corniger* (lab colony)
GH1	Glyco_hydro_1	PF00232	36	39	70	62
GH2	Glyco_hydro_2	PF00703	56	20	95	37
	Glyco_hydro_2_C	PF02836	96	39	154	56
	Glyco_hydro_2_N	PF02837	88	28	142	48
GH3	Glyco_hydro_3	PF00933	148	121	268	205
	Glyco_hydro_3_C	PF01915	100	48	165	94
GH4	Glyco_hydro_4	PF02056	71	28	106	40
	Glyco_hydro_4C	PF11975	59	21	92	48
GH5	Cellulase	PF00150	149	197	258	341
GH6	Glyco_hydro_6	PF01341	0	0	0	0
GH7	Glyco_hydro_7	PF00840	0	0	0	0
GH8	Glyco_hydro_8	PF01270	21	37	37	80
GH9	Glyco_hydro_9	PF00759	63	68	104	138
GH10	Glyco_hydro_10	PF00331	141	126	225	208
GH11	Glyco_hydro_11	PF00457	50	52	110	94
GH12	Glyco_hydro_12	PF01670	0	0	0	0
GH13	Alpha-amylase	PF00128	197	134	365	244
GH14	Glyco_hydro_14	PF01373	1	0	2	0
GH15	Glyco_hydro_15	PF00723	2	0	4	0
GH16	Glyco_hydro_16	PF00722	10	7	18	9
GH17	Glyco_hydro_17	PF00332	0	0	0	0
GH18	Glyco_hydro_18	PF00704	30	32	47	53
GH19	Glyco_hydro_19	PF00182	0	0	0	0
GH20	Glyco_hydro_20	PF00728	31	32	57	51
	Glyco_hydro_20b	PF02838	2	5	6	8
GH22	Lys	PF00062	0	0	0	0
GH23	SLT	PF01464	88	68	175	144
GH24	Phage_lysozyme	PF00959	3	0	5	0
GH25	Glyco_hydro_25	PF01183	7	1	8	1
GH26	Glyco_hydro_26	PF02156	18	26	34	58
GH27	Melibiase	PF02065	30	15	51	27
GH28	Glyco_hydro_28	PF00295	13	4	28	4
GH29	Alpha_L_fucos	PF01120	47	26	81	35
GH30	Glyco_hydro_30	PF02055	24	24	28	31
GH31	Glyco_hydro_31	PF01055	100	29	167	52
GH32	Glyco_hydro_32C	PF08244	4	0	5	0
	Glyco_hydro_32N	PF00251	14	1	23	1
GH33	BNR	PF02012	20	5	101	25
GH34	Neur	PF00064	0	0	0	0
GH35	Glyco_hydro_35	PF01301	3	1	3	1
GH36	BLAST search[c]		26	8	43	17
GH37	Trehalase	PF01204	15	8	30	20
GH38	Glyco_hydro_38	PF01074	22	11	33	15
	Glyco_hydro_38C	PF07748	17	13	18	18
GH39	Glyco_hydro_39	PF01229	24	15	41	28
GH42	Glyco_hydro_42	PF02449	27	37	41	57
	Glyco_hydro_42C	PF08533	3	6	7	7

GH ID	Pfam HMM Name	Pfam Accession	Gene Count[a]		Estimated gene copy[b]	
			A. wheeleri (cow dung)	*N. corniger* (lab colony)	*A. wheeleri* (cow dung)	*N. corniger* (lab colony)
	Glyco_hydro_42M	PF08532	15	21	22	35
GH43	Glyco_hydro_43	PF04616	143	58	252	104
GH44	BLAST search		5	0	7	0
GH45	Glyco_hydro_45	PF02015	14	33	20	71
GH46	Glyco_hydro_46	PF01374	0	0	0	0
GH47	Glyco_hydro_47	PF01532	1	0	2	0
GH48	Glyco_hydro_48	PF02011	1	0	1	0
GH49	Glyco_hydro_49	PF03718	0	0	0	0
GH50	BLAST search		8	0	16	0
GH51	BLAST search		53	9	102	16
GH52	Glyco_hydro_52	PF03512	0	9	0	22
GH53	Glyco_hydro_53	PF07745	13	35	17	64
GH54	ArabFuran-catal	PF09206	1	0	1	0
GH55	BLAST search		2	0	5	0
GH56	Glyco_hydro_56	PF01630	0	0	0	0
GH57	Glyco_hydro_57	PF03065	43	37	77	88
GH58	BLAST search		3	0	5	0
GH59	Glyco_hydro_59	PF02057	1	0	1	0
GH61	Glyco_hydro_61	PF03443	0	0	0	0
GH62	Glyco_hydro_62	PF03664	1	0	1	0
GH63	Glyco_hydro_63	PF03200	1	0	1	0
GH64	BLAST search		0	0	0	0
GH65	Glyco_hydro_65C	PF03633	8	1	12	1
	Glyco_hydro_65m	PF03632	41	3	65	3
	Glyco_hydro_65N	PF03636	13	2	27	2
GH66	BLAST search		4	0	4	0
GH67	Glyco_hydro_67C	PF07477	19	13	24	21
	Glyco_hydro_67M	PF07488	24	9	33	18
	Glyco_hydro_67N	PF03648	5	0	6	0
GH68	Glyco_hydro_68	PF02435	0	0	0	0
GH70	Glyco_hydro_70	PF02324	0	0	0	0
GH71	Glyco_hydro_71	PF03659	0	0	0	0
GH72	Glyco_hydro_72	PF03198	0	0	0	0
GH73	Glucosaminidase	PF01832	29	13	49	19
GH74	BLAST search		13	16	21	28
GH75	Chitosanase	PF07335	0	0	0	0
GH76	Glyco_hydro_76	PF03663	5	0	6	0
GH77	Glyco_hydro_77	PF02446	126	58	195	109
GH78	Bac_rhamnosid	PF05592	29	7	48	8
GH79	Glyco_hydro_79n	PF03662	0	1	0	1
GH80	BLAST search		0	0	0	0
GH81	Glyco_hydro_81	PF03639	0	0	0	0
GH82	BLAST search		0	0	0	0
GH83	HN	PF00423	0	0	0	0
GH84	NAGidase	PF07555	0	0	0	0
GH85	Glyco_hydro_85	PF03644	1	0	1	0
GH86	BLAST search		0	0	0	0
GH87	BLAST search		0	0	0	0
GH88	Glyco_hydro_88	PF07470	58	19	89	25

GH ID	Pfam HMM Name	Pfam Accession	Gene Count[a]		Estimated gene copy[b]	
			A. wheeleri (cow dung)	*N. corniger* (lab colony)	*A. wheeleri* (cow dung)	*N. corniger* (lab colony)
GH89	NAGLU	PF05089	0	2	0	3
GH90	BLAST search		0	0	0	0
GH91	BLAST search		0	0	0	0
GH92	Glyco_hydro_92	PF07971	9	7	14	7
GH93	BLAST search		0	0	0	0
GH94	BLAST search		74	52	125	118
GH95	BLAST search		29	11	42	19
GH96	BLAST search		0	0	0	0
GH97	Glyco_hydro_97	PF10566	5	9	7	14
GH98	Glyco_hydro_98M	PF08306	0	0	0	0
	Glyco_hydro_98C	PF08307	1	0	1	0
GH99	BLAST search		0	1	0	1
GH100	Invertase_neut	PF04853	0	0	0	0
GH101	BLAST search		0	0	0	0
GH102	MltA	PF03562	1	2	2	4
GH103	BLAST search		3	2	5	3
GH104	See GH24	PF00959				
GH105	See GH88	PF07470				
GH106	BLAST search		7	2	10	2
GH107	BLAST search		3	0	3	0
GH108	DUF847	PF05838	6	0	8	0
GH109	BLAST search		15	5	25	7
GH110	BLAST search		0	0	0	0

[a] Gene count is the number of genes in the metagenome.

[b] Estimated gene copy is the count of genes weighted by their individual read depth, which should reflect individual population abundance.

[c] BLAST search: corresponding CAZy is not available, and the identification is through Blastp search, with an e-value cutoff of 1e-6.
