# Supplementary material for: Comparative Metagenomic and Metatranscriptomic Analysis of Hindgut Paunch Microbiota in Wood- and Dung-Feeding Higher Termites
Source: PLoS One. 2013 Apr 12;8(4):e61126. doi: 10.1371/journal.pone.0061126 (PMC3625147; doi:10.1371/journal.pone.0061126)
Supplement: Table S7 — Estimated average genome size in metagenomes using single-copy phylogenetic markers (PDF) [file pone.0061126.s015.pdf]

Table S7. Estimated average genome size in metagenomes using single-copy phylogenetic markers [a]

COG ID	Function	Consensus length of each COG (bp)	Total bases in each COG (bp) [b]		Estimated copies of "full-length" genes in each COG [c]		Estimated average genome size (Mbp) [d]	
			A. wheeleri	*N. corniger*	*A. wheeleri*	*N. corniger*	*A. wheeleri*	*N. corniger*
COG0016	Phenylalanyl-tRNA synthetase alpha subunit	1005	72156	42929	72	43	3.6	3.3
COG0048	Ribosomal protein S12	387	14907	12194	39	32	6.7	4.5
COG0049	Ribosomal protein S7	444	20860	14017	47	32	5.5	4.5
COG0051	Ribosomal protein S10	312	15486	9195	50	29	5.2	4.8
COG0081	Ribosomal protein L1	684	41721	21326	61	31	4.2	4.5
COG0087	Ribosomal protein L3	654	31701	15326	48	23	5.3	6.0
COG0088	Ribosomal protein L4	642	31314	22193	49	35	5.3	4.1
COG0089	Ribosomal protein L23	282	11709	8570	42	30	6.2	4.6
COG0090	Ribosomal protein L2	825	46379	20489	56	25	4.6	5.7
COG0092	Ribosomal protein S3	699	42385	24971	61	36	4.2	4.0
COG0093	Ribosomal protein L14	366	21727	15002	59	41	4.3	3.4
COG0094	Ribosomal protein L5	540	40881	19266	76	36	3.4	4.0
COG0096	Ribosomal protein S8	396	25605	8233	65	21	4.0	6.8
COG0097	Ribosomal protein L6P/L9E	534	31501	14005	59	26	4.3	5.4
COG0098	Ribosomal protein S5	543	26419	21726	49	40	5.3	3.5
COG0099	Ribosomal protein S13	363	15655	13683	43	38	5.9	3.7
COG0100	Ribosomal protein S11	387	21411	9552	55	25	4.6	5.7
COG0102	Ribosomal protein L13	444	27399	9118	62	21	4.2	6.9
COG0150	Phosphoribosylaminoimidazole (AIR) synthetase	1035	60074	34455	58	33	4.4	4.2
COG0197	Ribosomal protein L16/L10E	438	24413	12272	56	28	4.6	5.0
COG0200	Ribosomal protein L15	456	23668	11238	52	25	4.9	5.7
COG0211	Ribosomal protein L27	261	12693	6237	49	24	5.3	5.9
COG0216	Protein chain release factor A	1089	67545	39011	62	36	4.1	3.9
COG0222	Ribosomal protein L7/L12	372	18070	14900	49	40	5.3	3.5
COG0256	Ribosomal protein L18	375	16164	9917	43	26	5.9	5.3
COG0292	Ribosomal protein L20	354	16018	8515	45	24	5.7	5.9
COG0504	CTP synthase (UTP-ammonia lyase)	1599	110962	68072	69	43	3.7	3.3
COG0541	Signal recognition particle GTPase	1353	65094	50637	48	37	5.3	3.8
COG0556	Helicase subunit of the DNA excision repair complex	1989	134297	64365	68	32	3.8	4.4
COG0632	Holliday junction resolvasome, DNA-binding subunit	603	34350	19688	57	33	4.5	4.3
COG0691	tmRNA-binding protein	459	22947	10636	50	23	5.1	6.1
**Median**							**4.6**	**4.5**

[a]Single-copy phylogenetic marker genes were used to estimate average genome size in metagenomes. Using the median of results obtained from all individual phylogenetic marker COGs listed above should provide a closer estimation than results obtained using a single COG.
[b]Total bases in each COG was estimated by summing up the lengths of all genes in each COG, and adjusted by read depths of individual genes.
[c]Estimated copies of "full-length" gene = total bases in one COG / its consensus COG length
[d]Estimated average genome size = total bases used in metagenome assembly / estimated copies of "full-length" genes in each phylogenetic marker COG
